# Nanoarchitecting intelligently encapsulated designs for improved cancer therapy

**DOI:** 10.3389/fbioe.2025.1587178

**Published:** 2025-05-01

**Authors:** Ying-Tong Ye, Hong-Ying Xia, Jie Li, Shi-Bin Wang, Ai-Zheng Chen, Ranjith Kumar Kankala

**Affiliations:** ^1^ Institute of Biomaterials and Tissue Engineering, Huaqiao University, Xiamen, China; ^2^ College of Chemical Engineering, Huaqiao University, Xiamen, China; ^3^ Fujian Provincial Key Laboratory of Biochemical Technology (Huaqiao University), Xiamen, China

**Keywords:** nanoencapsulation, surface engineering, biomembranes, phase-change materials, anti-cancer

## Abstract

Despite the success in exploring various aspects of origination and therapeutic strategies, cancer has remained one of the most dreadful metabolic disorders due to failure to eradicate tumors comprehensively and frequent recurrence because of acquired resistance to the drugs. Recently, several advancements have been evidenced in the fabrication of various smart nanocarriers encapsulated with multiple components. Several reasons for smart nanoencapsulation include the enhancement of the bioavailability of drugs, precise targetability to reduce adverse effects on normal cells, and the ability to enable controlled drug release rates at the tumor sites. In addition, these smart nanocarriers protect encapsulated therapeutic cargo from deactivation, responsively delivering it based on the physiological or pathological characteristics of tumors. In this review, we present various smart approaches for cancer therapy, including organic materials, inorganic components, and their composites, as well as biomembrane-based nanoencapsulation strategies. These nanoencapsulation strategies, along with practical applications and their potential in cancer treatment, are discussed in depth, highlighting advantages and disadvantages, as well as aiming to reveal the ultimate prospects of nanoencapsulation in enhancing drug delivery efficiency and targeted cancer therapy.

## Graphical Abstract

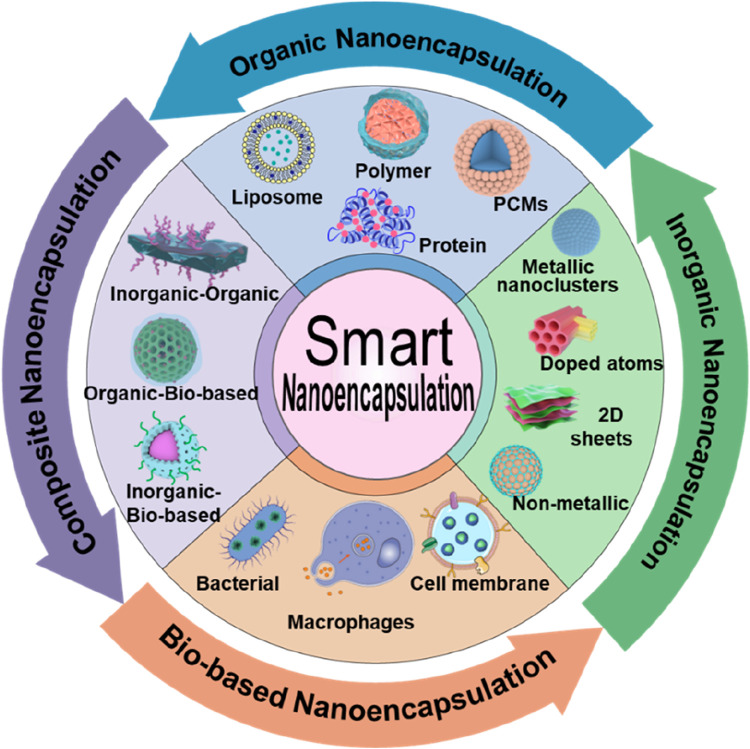

## 1 Introduction

Despite several efforts towards exploring the origination and developing various therapeutic modalities, cancer has emerged as one of the most dreadful metabolic diseases globally, second to cardiovascular diseases, accounting for millions of recorded cases every year (Siegel et al., 2023). Several established treatment options have included conventional surgical and radiotherapeutic interventions, as well as obligatory chemotherapy (Ji et al., 2018; Duan et al., 2023). Although these conventional treatment options showed favorable results to a considerable extent, this complex metabolic disorder often led to recurrence due to abnormal cell proliferation, affecting the quality of human life (Wang X. et al., 2021; Zhang Q. et al., 2022; Hao et al., 2023). In this regard, follow-up chemotherapy, various small-molecular active pharmaceutical ingredients (APIs/drugs), and immune boosters are inevitable, leading to an improved 5-year survival rate (Kankala et al., 2022). Nevertheless, the applicability of drugs often leads to various shortcomings, such as poor bioavailability, acquired multi-drug resistance (MDR), and adverse effects on normal cells, leading to poor therapeutic outcomes and requiring high therapeutic doses (Kankala et al., 2020a; Kankala et al., 2020b; Duan et al., 2023). Accordingly, various advancements have resulted in the fabrication of diverse nanoparticle-based composites, offering improved encapsulation efficacy due to a high surface-to-volume ratio and precise conveyance through overcoming tight barriers. Considerably, various smart, innovative architectures that can encapsulate therapeutic cargo are required to improve cancer therapy.

Since the advent of nanotechnology in the mid-1950’s, designing ultra-small nanoconstructs has garnered enormous interest from researchers in the development of various smart architectures using diverse synthesis methods and their advanced methodologies. Typically, nanoparticle-based delivery systems offer two major attributes such as abundant surface chemistry and a high surface-to-volume ratio (Mitchell et al., 2021). In this context, the guest drugs can be encapsulated within the nano-sized architectures, either porous or non-porous, through electrostatic (Peng et al., 2022) or van der Waals interactions (Wang X.-Y. et al., 2013) and immobilized by conjugating on their surfaces (Phan et al., 2022). Moreover, the ultra-small-sized containers specifically increase their accumulation in the tumors through leaky vasculature due to the enhanced permeation and retention (EPR) effect (Kankala et al., 2020b; Kang et al., 2020; Chen et al., 2023). Along this line, several advancements have been evidenced in the fabrication of diverse intelligent nanoarchitectures that can precisely respond to different stimuli, i.e., internal (pH and molecular) and external (light and magnetic) (Liu C. et al., 2019; Wang S. et al., 2023; Kankala et al., 2020a). The mild pH is the typical internal stimulus of the tumor environment, in which several materials are susceptible to mild pH, resulting in their disassembly towards unloading the therapeutic cargo, specifically in tumors (Ju et al., 2022; Kang et al., 2020). Similarly, the over-expressed glutathione (GSH) (Li et al., 2023; Shi et al., 2024) and hydrogen peroxide (H_2_O_2_) (Xu et al., 2023; Yu et al., 2017) levels act as internal molecular triggers improving the degradation of the carriers and generation of highly toxic radicals responsively towards improved ablation of tumor cells over the normal cells. Over the past few decades, several efforts have been dedicated to developing various kinds of multifunctional platforms for integrating numerous tumor treatment modes based on these different stimuli as triggers (Chen et al., 2023; Xia H.-Y. et al., 2022). However, the most direct measure to evaluate the treatment effect is to visually present the treatment effect by measuring the volume and weight of solid tumors. Comparatively, effective synergistic therapy using both external and internal triggers can usually achieve a 1 + 1 > 2 effect, which is superior to a single treatment modality (Bindra et al., 2021). Moreover, histological analysis also provides specific parameters for quantitative evaluation in clinical practice, such as TUNEL staining to detect apoptotic cells and the Ki-67 proliferation index, which can directly reflect the ability of synergistic therapy to inhibit tumor cell proliferation (Li D. et al., 2020; Gao et al., 2024). Specifically, in the scenarios of light-based treatment modalities, photothermal therapy (PTT) needs to monitor the local temperature rise of tumors in real time for a certain period to evaluate the photothermal conversion efficiency of nanomedicines. By detecting the level of reactive oxygen species (ROS, such as singlet oxygen) generation in tumor cells, the therapeutic efficiency of photodynamic therapy (PDT) can be reflected. The PTT-based active agents transform the light with deep penetration ability to localize the generation of heat, specifically in tumors, after their intratumoral accumulation (Li X. et al., 2020; Huang et al., 2013; Gao et al., 2012). Accordingly, these stimuli-responsive nanoparticle-based dosage forms show improved tumor-targeted efficacy while minimizing damage to healthy cells due to their unique size effects and adjustable physical and chemical properties, transforming current cancer diagnosis and treatment methods.

Despite the success in exploring the therapeutic potential of diverse nanoparticles, the designed nanoparticles, in many instances, fail to enhance therapeutic outcomes significantly compared to free drug counterparts (Huang et al., 2023). The limited therapeutic efficacy is largely attributed to various biological barriers, hindering their performance *in vivo* (Qin et al., 2023). Among various physicochemical properties influencing their behavior *in vivo*, the surface characteristics of the designed nanoparticles are particularly important, playing a decisive role in determining bioavailability, circulation time, and therapeutic effectiveness (Yan et al., 2023; Chen et al., 2021). To address these challenges, extensive research has focused on various functionalization strategies aimed at modifying the surfaces of nanoparticles to optimize cancer theranostics (Mei et al., 2022). Various smart nanoencapsulation approaches encapsulating drugs and nanoparticles are particularly important in cancer therapy for several predominant reasons. Typically, these smart nanoencapsulation strategies improve the encapsulation efficiency of drugs within their containers and on the surface. Owing to the strong interactions between the host and guest species, the undesirable wastage of drugs is significantly reduced during the conveyance in the physiological fluids. Nanoencapsulation can enhance the stability and circulation of nanoparticles by protecting them from immune recognition and clearance (Perciani et al., 2021). Furthermore, various targeting moieties can be attached to their surface to achieve targeted therapy and imaging, thereby enhancing active targeting capabilities toward specific tissues or cells of interest (Wang X. et al., 2023). The precise targeting ability of these intelligent materials offers significant enhancement of the bioavailability of the encapsulated therapeutic cargo.

To achieve effective tumor-targeted drug delivery, nanoencapsulated drugs must exhibit relatively long systemic circulation and have good and regulated biodegradability. However, enhancing stability to prolong circulation may hinder the timely degradation and release of drugs. Thus, the solution to this conflicting issue is to design intelligent and responsive materials. Additionally, the surface of nanoparticles can be customized to enable controlled functionalities using various nanoencapsulation strategies. These strategies are designed to respond exclusively to specific internal triggers (pH, temperature, redox conditions, enzymes, or hypoxia) or external triggers (light or ultrasound) at precise sites of action. On the one hand, the internal stimuli-responsive system typically utilizes the pathological conditions inherent in the TME to stimulate the release behavior. For example, pH-responsive nanocarriers utilize the acidic environment of tumors to alter the conformation of the carrier, further inducing drug release and ensuring local activation (Panja et al., 2025). Although elevated GSH levels can activate the redox-sensitive system for the effective release of drugs (Chen et al., 2025), it may pose certain challenges in practical clinical applications due to differences between the anatomical and physiological characteristics of the patients, affecting the pharmacokinetic and pharmacodynamic features. Typically, TME is often in a state of hypoxia. Modifying hypoxia-responsive aptamers on the designed nanosystems can enhance the affinity of nanocarriers for tumors and reduce the off-target effects (Chen H. et al., 2022). On the other hand, external stimuli-based response systems activated by light, ultrasound, or magnetic fields provide an alternative strategy through external manipulation. Although their application is limited by tissue penetration depth, the light-responsive carriers can accurately regulate the spatial and temporal release of drugs. To improve light penetration by overcoming tissue barriers, ultrasound-responsive systems are commonly used for deep tissue activation response. However, the release efficiency depends on the acoustic power parameters applied. The magnetic-responsive nanocarriers can provide targeted delivery through external magnetic field guidance and control. These stimuli-responsive materials would offer controlled drug release rates, ensuring sustained action at the tumor site specifically and reducing adverse effects on normal cells. Moreover, these intelligent nanocarriers protect drugs from degradation and can be tailored to respond to the physiological or pathological characteristics of tumors.

**FIGURE 1:**
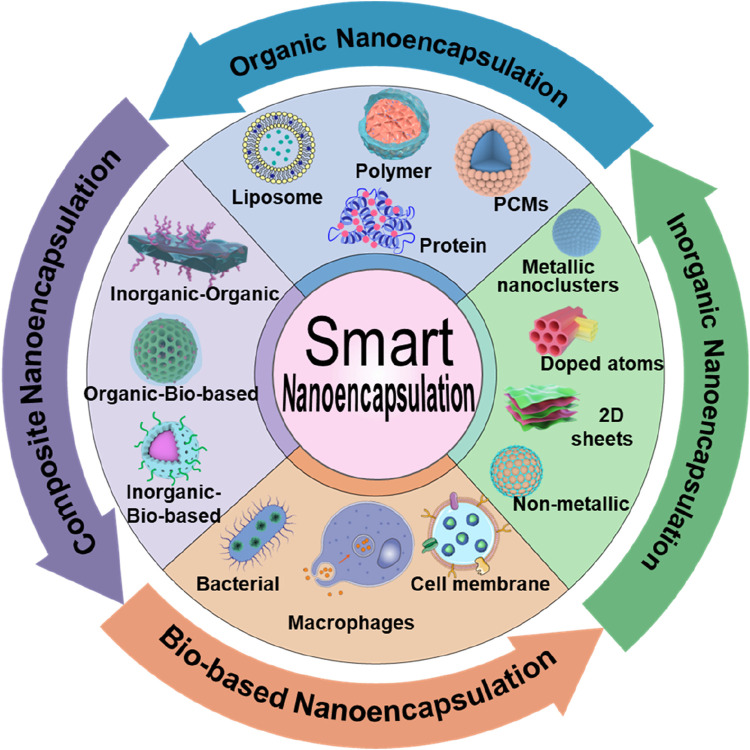
Schematic illustrating various compositions of smart nanoencapsulation strategies for cancer therapy.

In this review, we present discussions on intelligent nanoencapsulation technologies, categorizing the types of nanoencapsulation materials, including organic, inorganic, and composites, as well as biomembrane-based nanoencapsulation approaches (Figure 1; Table 1), highlighting their importance towards improved delivery of ultrasmall-sized nanoparticles or drugs for cancer therapy. Specifically, the recently emerged nanoencapsulation approach using biomembrane-based components, such as bacteria, cells, and miscellaneous composite membranes (macrophages and red blood cells, RBCs), is elaborated, exploring their pros and cons. Finally, we summarize the article with perspectives in terms of the advancement of new technologies, expecting them to provide personalized treatment options for cancer patients and significantly improve outcomes.

**TABLE 1:** A summary of various smart nanocarriers encapsulated with multiple components for cancer therapy.

Type of encapsulation		Material	Drug	Outcome	References
Organic	Polymer	Pt-MoSe_2_-PEG	—	The designed Pt-MoSe_2_-PEG with exceptional ROS and hyperthermia generation abilities presented synergistic trimodal (PTT/PDT/CDT) efficacies, showcasing great potential for cancer therapy.	Meng et al. (2024)
		PFV/CaCO_3_/PDA@PEG	—	A well-designed nanoparticle contributed to realizing PDT, mitochondrial dysfunction, and ROS-triggered Ca^2+^ overload synergistic therapy, displaying superior *in vivo* inhibition of 4T1 tumor growth and antimetastatic effect by both intravenous and intratumoral injection modes.	He et al. (2024b)
		HA-CuH-PVP	—	These composites showed the dispersity of reactants to increase reaction rates, offering hydrophilicity.	He et al. (2024a)
		HA-TPP/A	AMG510	The HA-TPP/A nanoparticles led to ubiquitination-dependent proteasomal degradation of mutp53 by targeting damage to mitochondria.	Mei et al. (2024)
	Liposome	Nano-Pt/VP@MLipo	Verteporfin	Oxygen produced at the tumor site by nano-Pt catalyzation improved the VP-mediated PDT, in turn triggering the release of nano-Pt via membrane permeabilization. The ultrasmall 3–5 nm nano-Pt enabled better penetration in tumors, facilitated by the generated oxygen gas, for enhanced chemotherapy.	Liu et al. (2021)
		Fe_3_O_4_@PGL/Fe_3_O_4_@Lipid nanoparticles	—	The Fe ions released *in vitro* directly contributed to the Fenton reaction. In contrast, the presence of RAW 264.7 macrophages accelerated the ROS generation and further induced increased cellular toxicity of HT-29 cancer cells.	Liu et al. (2022b)
	Protein	Albumin nanoparticles	Paclitaxel (PTX) and Fenretinide (4-HPR)	The albumin nanoparticles enhanced blood-brain barrier penetration/intratumoral infiltration/and cellular uptake.	Lin et al. (2016)
		BAY-876/V-9302@HSA nanoparticles	BAY-876/V-9302	These composites inhibited glucose and glutamine uptake of pancreatic cancer cells through the released BAY-876 and V-9302, leading to nutrition deprivation and oxidative stress, activating caspase 1 and GSDMD, and finally, inducing pyroptosis.	Wang et al. (2024a)
		ICG@HSA-C5 nanoparticles	ICG	These composites presented inhibition of cisplatin-resistant tumor growth, improving the targeting abilities.	Man et al. (2024)
		BSA-IrO_2_ nanoparticles	—	BSA-IrO_2_ nanoparticles could act as catalase to protect normal cells against H_2_O_2_-induced reactive oxygen pressure and inflammation while significantly enhancing photoacoustic imaging through microbubble-based inertial cavitation.	Zhen et al. (2018)
		ZnS@BSA	—	Intracellular zinc ions could produce reactive oxygen species, facilitated by the generated H_2_S gas from ZnS@BSA via specifically inhibiting catalase in hepatocellular carcinoma cells through activating the cGAS/STING signals in mice.	Cen et al. (2021)
	PCMs	PCM	IR780	These composites improved the therapeutic specificity and efficacy of coagulation-based tumor therapy.	Yin et al. (2021)
		PCM	PTX/IR780/GA	These PCM composites with photostability endowed with hyperthermia-triggered release/dampened the side effects.	Wang et al. (2023d)
		IR780-sMnO_2_-PCM nanoparticles	IR780	These PCM composites prevented IR780 from photodegradation/immediately released MnO_2_, enhancing the inhibition of tumor growth.	Zhang et al. (2019)
		DSF/Cu_2-x_Se@PCM	DSF	These composites safeguarded drug biocompatibility, significantly boosting drug-relative concentration at the tumor location.	Pan et al. (2024)
Inorganic	Non-metallic	GO-PEG-Cy5.5	DOX	The designed GO-PEG-Cy5.5 composites presented enhanced tumor uptake of the nanodrug toward effective drug delivery.	Nie et al. (2014)
		TZ@SWCNTs		The designed TZ@SWCNTs presented an enhancement of stability, kinetics, and biocompatibility/superior pharmacokinetics.	Li et al. (2020b)
		GOMs	Paclitaxel (PTX)	GOMs presented pH-dependent drug release.	Wei et al. (2021)
		LPSi nanoparticles		LPSi nanoparticles with intrinsic NIR photoluminescence enabled monitoring of both accumulation and degradation *in vivo* in a mouse model.	Park et al. (2009)
		PEI-MSN		The surface coating by PEI could accelerate the hydrolytic degradation of MSNs regardless of the pH value of the PBS medium.	Choi and Kim (2019)
		Janus-type nanoparticles	DOX	These Janus containers showed apoptosis-mediated cancer cell death after intracellular delivery of drug cargo.	Liu et al. (2019a)
		Ca/Cu-BG		These metal-doped composites presented exceptional anti-tumor effects in human osteosarcoma cells (HOS) through improved cellular internalization efficacy and free radical generation, respectively/confirmed Fenton-like reaction-induced chemodynamic therapy (CDT), over-expressed hypoxia-inducible factor (HIF)-1α through p53 pathway, and formation of calcified nodules.	Han et al. (2022)
	Metallic	DOX@MgAl-LDH	DOX	The MgAl-LDH composites exhibited excellent tumor targeting, enhanced cellular uptake, and cytotoxicity against cancer cells compared with free DOX, significantly inhibiting tumor growth with decreased DOX-induced cardiotoxicity compared with free DOX.	Hakeem et al. (2018)
		MgAl-LDHs	Camptothecin	The encapsulation method allowed for an approximately threefold increase in the solubility of camptothecin, suggesting the potential for cancer therapy.	Tyner et al. (2004)
		LDH-Gd/Au	DOX	The high specific surface area and the layered structure of LDH-Gd/Au with the high probability of water molecule access to the gadolinium ions doped in the LDH layer lattice showed the consequent significant water molecule relaxivity enhancement, essentially beneficial in enhancing MR imaging/favorable to CT imaging.	Wang et al. (2013a)
		FA-CM	Hydrophilic DOX and hydrophobic drug [paclitaxel (PTX)]	Versatile nanocarriers of DOX and PTX loaded on FA-CM with targeted ability and excellent biocompatibility could be applied in clinical practice and medical imaging.	Wen et al. (2019)
		HA-ZnO-PEG	DOX	The HA-ZnO-PEG system exhibited a response to acidic pH to trigger selective drug release in tumors and improved the therapeutic index.	Cai et al. (2016)
		ZnO-PG-RGD/DOX	DOX	ZnO-PG-RGD was selectively taken up by U87MG, not Hela cells, demonstrating an obvious targeting property.	Yang et al. (2019b)
		ZnO nanoparticles	DOX	As an immunomodulator, ZnO nanoparticles could effectively downregulate CD44, protect macrophages from DOX-induced toxicity, and boost the DOX-induced macrophage polarization toward an M1-like phenotype.	Wang et al. (2017), Zhang et al. (2011), Chen et al. (2019)
		BSArGO@ZIF-8 NSs		BSArGO@ZIF-8 NSs mediated ion-interference and photothermal combined therapy led to effective apoptosis and inhibited cell proliferation and angiogenesis, bringing a higher efficacy in tumor suppression *in vivo*.	Lv et al. (2022)
Bio-based	Bacterial	Non-pathogenic *Escherichia coli* strain	CD47nb	Delivery of CD47nb by tumor-colonizing bacteria increases activation of tumor-infiltrating T cells, stimulates rapid tumor regression, prevents metastasis, and leads to long-term survival in a syngeneic tumor model in mice.	Chowdhury et al. (2019)
		Dead EC-K1	ICG	The inactive Trojan EC-K1 could take therapeutics and bypass the BBB together after intravenous injection into the mice.	Lu et al. (2023)
	Cells as carriers	LD-MDS	Legumain-specific pro-peptide of melittin (legM)/cytotoxic soravtansine (DM4) prodrug	LD-MDS is responsively activated by legumain protease and converted into DM4-loaded exosome-like nanovesicles (DENs), facilitating efficient internalization by metastatic 4T1 cancer cells and considerable cell death.	Cao et al. (2018)
		M-SMN	SMA-AANK-Mertansine	The anticancer drugs are intelligently released from M-SMNs as free drug molecules and drug-loaded microvesicles, resulting in considerable inhibition of the proliferation, migration, and invasion activities of metastatic 4T1 breast cancer cells/M-SMNs significantly improve the delivery to lung metastases and penetrate the metastatic tumors, thus producing a 77.8% inhibition of lung metastases.	He et al. (2017)
		Neutrophil		The specific type of nanoparticles largely determined their behavior in blood vessels and their neutrophil-mediated delivery to the tumor. Since neutrophils are the first to migrate to the site of inflammation, they can increase nanodrug delivery effectiveness for nanomedicine.	Garanina et al. (2023)
	Cell membrane	RBC-ENPs		RBC membrane-coated elastic PEGDA hydrogel nanoparticles (RBC-ENPs) exhibited high immunocompatibility and achieved excellent diffusion in the tumor ECM, leading to improved multicellular spheroid penetration and tumor tissue accumulation.	Miao et al. (2022)
		RBC@ MMSNs		RBC@MMSNs could avoid immune clearance and achieve magnetic field (MF)-induced high accumulation in a tumor, rapidly generating singlet oxygen from loaded hypocrellin B (HB) and leading to tumor necrosis.	Xuan et al. (2018)
		TNBC cell membranes	Programmed death-ligand 1 (PD-L1) inhibitors	Homologous targeting and camouflage properties endowed the nanodelivery system with excellent biocompatibility, demonstrating excellent synergistic therapeutic efficacy.	Xiong et al. (2024)
Composite nanoencapsulation		DOX@CaCO_3_/mHA-PEG nanoparticles	DOX	The released DOX in MCF-7 and MDA-MB-231 cells migrated into the cell nucleus, while the released Ca^2+^ moved outside the cells to induce tumor vessel coagulation.	Zhou et al. (2023)
		MSN-hyd-MOP	DOX	The increased acidity in endo-/lysosome promoted DOX-loaded MSN-hyd-MOP (MSN-hyd-MOP@DOX) and released DOX payload quickly to tumor cells.	Yuan et al. (2019)
		CaCO_3_@PEI		The intracellular proton sponge effect caused by PEI and CO_2_ by the CaCO_3_ nanoparticles not only facilitated nanomotors to escape from the lysosomes toward the cytoplasm but also promoted the deep penetration and long-time retention of nanomotors inside tumor cells.	Ren et al. (2022)
		^PEG^CaNM_CUR+CDDP_	Cisplatin (CDDP)/curcumin (CUR)	The PEGylated CaNM_CUR+CDDP_ (^PEG^CaNM_CUR+CDDP_) selectively accumulated in tumor tissues and induced multilevel destruction of mitochondria by the combined effects of burst Ca^2+^ release, Ca^2+^ efflux inhibition by CUR, and chemotherapeutic CDDP, thereby observably boosting mitochondria-targeted tumor inhibition.	Zheng et al. (2021)
		M@CaCO_3_@KAE	Kaempferol-3-O-rutinoside (KAE)	CaCO_3_@KAE specifically responded to TME and consequently released KAE and calcium ions. Accordingly, the KAE-mediated calcium overload destructed mitochondrial structure and functions, causing cytoskeleton collapse and oxidative stress, as well as leading to cancerous cellular apoptosis.	Li et al. (2021)
		PEG-cZnO QDs	DOX	The ZnO QDs exhibited cytotoxicity post-dissolution and preferentially killed cancerous cells compared to normal cells.	Cai et al. (2017)
		H-MnO_2_-PEG/C&D	Chlorine e6 (Ce6)/DOX	Hollow H-MnO_2_ nanoshells could modulate the tissue microenvironment, releasing a drug and inhibiting tumor growth alone or in combination with checkpoint blockade therapy.	Yang et al. (2017)
		Lipo/HMME/ACF@MnO_2_-AS1411	Ematoporphyrin monomethyl ether (HMME) and acriflavine (ACF)	The Lipo/HMME/ACF@MnO_2_-AS1411 delivery system showed codelivery of HMME and ACF, pH/GSH/ultrasound triple responses, synergistic cascaded enhancement of SDT, precise tumor-targeting, and imaging.	Wang et al. (2018)
		ZnO quantum dots (QDs)-gated MSNs	DOX	ZnO QDs presented as a dual-purpose entity that not only acted as a lid but also showed a synergistic antitumor effect on cancer cells.	Muhammad et al. (2011)
	MOFs	5-FU@GA-MOFs	5-FU	These composites presented increased cytotoxicity towards HepG2 cells/increasing the accumulation of 5-FU/liver targeting ability.	Li et al. (2020c)
		PMOF@AuNP/hairpin		The PMOF@AuNP/hairpin nanotheranostic showed catalase activity to catalyze H_2_O_2_ to O_2_ to ameliorate tumor hypoxia and thus enhance the PDT effect.	Yang et al. (2023)
		Nic-MOF@HA	Nicorandil (Nic)	MOFs offered high loading efficiency, converting the oxygen into cytotoxic ROS.	Xia et al. (2022b)
		TPZ/UCSs	Tirapazamine (TPZ)	The designed MOFs exhibited synergistic PDT/hypoxia-activated chemotherapy/immunotherapy/high loading efficiency and content of PSs.	Shao et al. (2020)
		Al-MOF	OVA	The Al-MOFs showed resistance to ambient temperature and pH, acting synergistically as a delivery vehicle synthesizing an adjuvant over a model antigen ovalbumin (OVA) to act as an armor.	Miao et al. (2019)

## 2 Organic nanoencapsulation

Although different kinds of nanoparticle-based delivery systems offer numerous advantages in cancer therapy, the biological behavior of these innovative delivery systems *in vivo* is influenced by various factors due to the complexity of the physiological environment. These factors include barriers posed by biological membranes and clearance mechanisms of the immune system, hindering the precise targeting efficacy and release of therapeutic cargo. Typically, most nanoparticle-based delivery platforms rely on passive targeting of tumor cells that depend solely on the EPR effect, requiring precise targeting of solid tumors. Organic-based nanodelivery systems offer significant advantages, such as excellent biocompatibility and high drug-loading efficiency. However, there are still some limitations in the clinical application of these biocompatible. In addition, the dense extracellular matrix (ECM) and hypoxic microenvironment in deep-seated solid tumors limit drug penetration and uniform distribution, thereby reducing overall therapeutic efficacy. Furthermore, these systems are often rapidly cleared by macrophages, further diminishing the effective delivery of encapsulated drugs. Nonetheless, recent advancements have been evidenced in the development of functionalized smart organic-based materials, which are being developed to gradually overcome these shortcomings. Consequently, encapsulating nanodelivery systems with functionalized smart organic-based materials can not only improve their colloidal stability in physiological fluids and shield against macrophage clearance by effectively “gate-keeping” the encapsulated cargo but also confer enhanced targeting capabilities toward tumors (Table 1).

### 2.1 Polymers

**FIGURE 2:**
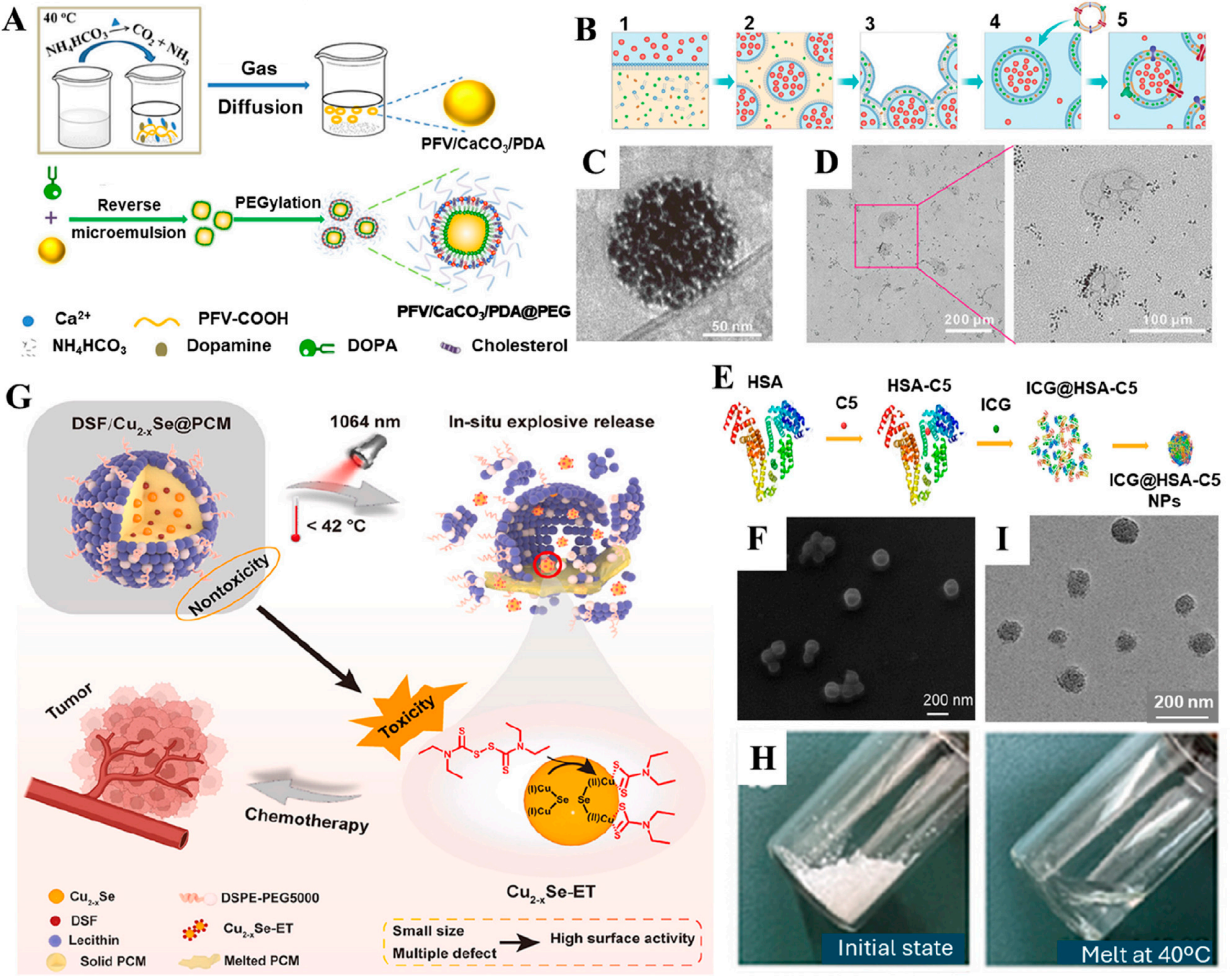
Various organic-based nanoencapsulation strategies. **(A)** Schematic illustration of the preparation and PEGylation process of PFV/CaCO_3_/PDA@PEG nanoparticles. Reproduced with permission from Ref. (He J. et al., 2024) Copyright 2024, American Chemical Society. **(B)** Schematic illustration of the fabrication of nano-Pt/VP@Mlipo. **(C)** An enlarged Cryo-TEM image of nano-Pt/VP@MLipo. **(D)** TEM observation of the light (690 nm) irradiation-triggered nano-Pt liberation from the aqueous cavity of nano-Pt/VP@MLipo. Reproduced with permission from Ref. (Liu et al., 2021) Copyright 2021, John Wiley & Sons. **(E)** Preparation scheme of ICG@HSA−C5 nanoparticles. **(F)** SEM image of ICG@HSA−C5 nanoparticles. Reproduced with permission from Ref. (Man et al., 2024) Copyright 2024, Springer Nature. **(G)** Schematic illustration of the NIR-II light-triggered nanoreactor as an in situ ultrafast Cu_2-x_Se-ET generator for precise cancer chemotherapy. **(H)** The PCM undergoes a transition from solid state (upper) to liquid state (bottom) as it reaches a temperature of 40°C. **(I)** TEM of DCP. Reproduced with permission from Ref. (Pan et al., 2024) Copyright 2024, KeAi Publishers.

Several materials with organic backbones have been explored to fabricate nanoparticle-based delivery systems. Typically, these supramolecular assemblies offer exceptional attributes, such as tailorable physicochemical features, optimal morphological characteristics, biocompatibility, and degradability. These polymeric assemblies have been employed to conceal nanoparticle “foreign bodies” from the immune system as stealth coatings, including polyethylene glycol (PEG), polyvinylpyrrolidone (PVP), polyvinyl alcohol (PVA), and hyaluronic acid (HA). In addition, these components significantly enrich the stability and permeability of nanocarriers, facilitating improved bioavailability of drugs and their antitumor efficacy. In addition to developing nanoconstructs, various other kinds of nanocarriers can be modified with hydrophilic PEG segments to precisely target the tumor, achieving not only strong responsiveness but also improving biocompatibility and enhancing the stability of nanocarriers in the systemic circulation (Meng et al., 2024). In addition, PEG offers aqueous solubility, stabilizing the structure of nanocarriers after synthesis and effectively preventing the undesired clearance of nanoparticles by the mononuclear phagocyte system in the physiological fluids (Figure 2A) (He J. et al., 2024). PVP is another biocompatible polymer often preferred for encapsulating various kinds of drugs and nanoparticles owing to various characteristics, including enhancement of the dispersibility and biocompatibility of nanodelivery platforms. Despite the efficacy, these polymeric carriers suffer from several limitations, such as failure to target the tumor microenvironment (TME) and deprived degradability in the tumors, resulting in the poor bioavailability of drugs. Nevertheless, some polymers can address these limitations, such as HA. Since the ECM of the TME contains abundant hyaluronidase, encapsulating nanodelivery carriers with an HA shell allows the nanocarriers to be degraded by hyaluronidase at the tumor site, resulting in accumulation at the tumor and increased drug delivery efficiency. HA is not only highly reactive due to functional groups like acetamide, aldehyde, carboxyl, and hydroxyl but also targets the overexpressed CD44 receptors commonly found in various tumors (Phua et al., 2019). Along this line, several reports demonstrated that encapsulating nanocarriers or drugs with an HA shell not only improved the tumor-targeting accuracy (Wang W. et al., 2022; Huang et al., 2022) of the nanodelivery system but also enhanced its dispersibility (He G. et al., 2024).

Despite the success in fabricating nanoparticles using biodegradable polymers, it is highly challenging to eradicate tumors using a single polymer-coated nanodelivery system due to the complexity of malignant tumors, failing to reach the tumor site and ablate the cells effectively. Consequently, several efforts have been dedicated to exploring various advancements in terms of developing dual-targeting coatings for various tumor markers. For instance, He and colleagues fabricated a dual delivery platform, which was initially functionalized with PVP and then encapsulated with an HA shell, proposing a new approach for targeting deep-seated tumors. In this approach, PVP not only improved the aqueous dispersibility and stability of the nanoparticles but also ensured a controlled, pH-responsive release of bioactive Cu^+^ ions and hydrogen under the typical acidic conditions of the TME. Concurrently, the HA coating provided active tumor targeting through specific binding to CD44 receptors. Due to the overexpression of CD44 receptors on cancer cells, these designed composites thereby facilitated preferential accumulation within malignant tissues (He G. et al., 2024). In another case, Mei and colleagues encapsulated nanocarriers within functionalized HA-TPP conjugates. This smart design not only targeted mitochondrial damage, leading to ubiquitin-dependent proteasomal degradation of mutp53 but also precisely targeted gastrointestinal cancers (Mei et al., 2024). In addition to the common polymer encapsulation systems, dendrimer-based macromolecules possess precise internal cavities and a large number of surface functional groups, which can be easily functionalized and used as an ideal nanodrug delivery system. For example, Gao and coworkers constructed MnO_2_-embedded G5 PAMAM dendrimers and partially modified them with MPEG and PBA for coding delivery of GOx and cGAMP. With the continuous release and accumulation of Mn^2+^ in the TME, local starvation of tumors could trigger ROS generation, promoting immunogenic cell death (ICD) and damage-associated molecular patterns (DAMPs) of cancer cells for systemic immune activation and thereby improving the therapeutic effect of tumors. The experimental results showed that this nanocarrier showed an inhibitory effect on distal tumors (Gao et al., 2023). Together, these innovative polymer-based nanomaterials with smart designs demonstrated several advantages, such as extended circulation in the bloodstream (Cao et al., 2020), reduced drug leakage (Wang J. et al., 2021), controlled release, and precise targeting, among others (You et al., 2024). Despite the success, several aspects must be addressed comprehensively, requiring extensive investigations. These include reproducibility and colloidal stability on a large scale, biological safety at a genomic level, and precise therapeutic efficacy in natural tumors. Accordingly, there is an enormous scope, and it is expected to become a novel strategy in the fight against tumors soon.

### 2.2 Liposomes

Liposomes are referred to as replicas of cell membranes due to their hollow-like biomimetic vesicles enclosed by a lipid bilayer (Wang X. et al., 2022). These innovative structures offer broad-spectrum drug loading capacity, prolonged circulation *in vivo*, and excellent biocompatibility, making them one of the most successful nanodrug delivery vehicles to date (Kumar, 2019). Due to their unique lipid bilayer structure, liposomes can encapsulate both hydrophobic small molecules and hydrophilic macromolecules (Cao et al., 2020; Filipczak et al., 2020). The encapsulation of drugs in liposomal layers enhances the stability of drugs and their nanoparticulate forms, significantly improving their prolonged circulation and subsequent bioavailability in tumors, resulting in more efficient delivery of drugs compared to others. Considering these advantages, the United States Food and Drug Administration (US-FDA) has approved 20 liposome-related nanoparticle-based delivery systems for clinical cancer treatment, and 71 others are undergoing clinical trials. In a case, Liu and coworkers encapsulated nano-platinum (nano-Pt) in the inner aqueous cavity of liposomes using the reverse phase evaporation technique (Figures 2B–D). The designed liposomal nanoparticles permeabilized the membrane to deliver ultra-small Pt nanoparticles, achieving targeted delivery and deep tissue penetration in tumors (Liu et al., 2021). Combined with PDT, these nanocomposites exhibited exceptional tumor ablation. In another case, PEG-functionalized iron-based nanoparticles were embedded into the lipid bilayer (Liu Y. et al., 2022). Integrating amphiphilic PEG with the liposomal bilayer significantly enhanced the permeability of the cell membrane, increasing the production of ⋅OH, leading to lipid peroxidation and efficiently inducing ferroptosis in cancer cells. The delivery of therapeutic drugs into the bone microenvironment faces major challenges in treating osteosarcoma due to the compact bone structure and the low affinity between nanodrug carriers and bone tissue. The alendronate (ALN) strongly chelates osseous hydroxyapatite through calcium ions (Ca^2+^), which rapidly accumulate in the bone microenvironment. Considering the chelation ability of ALN, Chen and colleagues prepared a cellular drug delivery system based on ALN-containing liposomes loaded with an anti-osteosarcoma drug (methotrexate, MTX) for precise targeting of bone diseases, thereby achieving effective inhibition of osteosarcoma *in situ* (Chen et al., 2024). In addition, these smart, responsive liposomal formulations could be designed to react to specific stimuli in the TME, such as pH, temperature, or enzymes, enabling precise and controlled drug release (Fernandes et al., 2024). Considering the development of new technologies, liposome-based nanodrug encapsulation is poised to become a vital tool in future cancer therapies.

### 2.3 Proteins

Recently, significant progress has been evidenced by delivering small-molecule drugs using proteins to improve both the efficacy and safety during cancer therapy (Wang S. et al., 2023). As one of the representative members of biopolymers, protein-based nanoparticles offer several advantages, such as low toxicity, ease of functionalization, and high physiological stability. Predominantly, proteins have been applied for surface modification of various other kinds of nanocarriers, protecting them from premature enzymatic degradation and leakage while enhancing drug activity *in vivo* (Table 1) (Hong et al., 2020). Furthermore, the non-antigenic nature of proteins makes them suitable for targeted therapies. Interestingly, these proteins support the encapsulated cargo to maintain stable plasma colloid osmotic pressure. As a non-specific transport protein, albumin is the classic example of drug delivery, which not only acts as a carrier for exogenous substances but also protects from triggering autoimmune responses in the bloodstream. In addition, albumin can bind to albumin-binding receptors like Gp60 on the surface of tumor cells (Lin et al., 2016). Considering the source of extraction, the common types of albumin include human serum albumin (HSA), bovine serum albumin (BSA), and ovalbumin (OVA). In a case, Wang and colleagues developed HSA-based nanoparticles for delivering GLUT1 and ASCT2 inhibitors (BAY-876/V-9302@HSA nanoparticles) through a self-assembly process, proposing a nanodrug system based on bioinformatic analysis to combat malignant pancreatic cancer (Wang X. et al., 2024). Beyond self-assembly, HSA nanoparticles can also form complexes with metals and crosslink with anticancer drugs. For instance, Man and colleagues synthesized a platinum-based compound using BSA that showed significant cytotoxicity against cisplatin-resistant ovarian cancer cells (Figures 2E,F) (Man et al., 2024). Then, they constructed a dual drug delivery system encapsulating the photosensitizer indocyanine green (ICG), shortly referred to as ICG@HSA-C5 nanoparticles. To this end, BSA derived from animals is preferred over HSA due to various advantages, such as excellent biocompatibility, non-toxicity, and non-immunogenicity, as well as relatively low cost, among others. For example, Zhen and coworkers functionalized BSA on iridium oxide (IrO_2_) nanoparticles, endowing the nanodelivery platform with excellent photothermal conversion efficiency and peroxidase-like activity while enabling photoacoustic (PA)/computed tomography (CT)/photothermal imaging (PTI)-guided PTT against tumors (Zhen et al., 2018). In another instance, Chen and colleagues designed a nanocluster self-assembled from zinc sulfide (ZnS) and BSA, enhancing immunotherapy against liver cancer by activating the cyclic GMP–AMP and its receptor stimulator of interferon genes (cGAS/STING) signaling pathway in mice, promoting CD8^+^ T cell infiltration and dendritic cell cross-presentation at the tumor site (Cen et al., 2021). In addition, Yang and colleagues demonstrated that BSA as a scaffold ligand could retain the primary structural and biological functional groups of native BSA. Moreover, this scaffold provided sequestered-entrapped-reduction ability to Au (III), forming BSA-stabilized Au nanoclusters (Au NCs). These composites were successfully transported to the tumor site and responded to the TME (Yang et al., 2022). The current research also focuses on the utilization of human ferritin (HFN). This iron storage protein exists in human cells, possessing a unique core-shell structure. The shell is self-assembled by 24 subunits to form a protein cage, and the inner cavity can store iron in the form of ferrihydrite. Temperature-controllable small-molecule drug channels exist on the ferritin cage, endowing ferritin with excellent performance as a tumor-targeting drug carrier. Zhang and colleagues altered the mutation site of ferritin. They constructed nucleic acid-loaded ferritin nanocages using the self-assembly method for the delivery of Toll-like receptor (TLRs) agonistic nucleic acids, thus effectively inhibiting tumor growth and metastasis (Zhang B. et al., 2022).

### 2.4 Phase change materials (PCMs)

PCMs are constituents that can undergo phase transition within a specific temperature range (Farid et al., 2004). These materials are often characterized by high latent heat of fusion and exhibit reversible solid-liquid transformations over a small temperature range, making them highly interesting as thermos-responsive drug release carriers (Hyun et al., 2014). In general, it is necessary to finely regulate the composition of PCMs, whose melting point is higher than normal body temperature (about 45.5°C), ensuring the stability of the nanocarrier during cycling. In addition, targeted proteins or antibodies can be engineered on the surface of the PCM nanocarrier system. These engineered composites can improve targeting efficiency and distribution in the TME. Further, triggering the melting of the PCM layer through external stimulation can prevent premature phase transition and leakage of encapsulated nanoparticles due to complex temperature fluctuations in the body environment (Zou et al., 2022). PCMs are highly biocompatible, facilitating the incorporation of various functional nanomaterials or molecules into tightly encapsulated carriers and enabling rapid release of their payload under near-infrared (NIR) irradiation (Zhang et al., 2019; Yin et al., 2021; Zhu et al., 2021). Various PCM-based composites include thermo-responsive materials based on natural fatty acids (capric acid, CA, lauric acid, LA, stearic acid, SA, and octadecane, OD) or fatty alcohols (1-tetradecanol), as well as their eutectic mixtures. In recent years, PCM-based nanocomposites have attracted significant attention from researchers, particularly in encapsulating PTT-based nanoparticles or molecular photosensitizers. For example, Yin and colleagues co-encapsulated thrombin (Thr) with photothermal properties and the photosensitizer IR780 within a PCM matrix (Yin et al., 2021). Similarly, Wang and coworkers designed PCM-based composites co-encapsulated with paclitaxel (PTX), IR780, and gambogic acid (GA) (Wang Y. et al., 2023). In another instance, Zhang and coworkers loaded IR780 and ultra-small manganese dioxide (MnO_2_) nanoparticles into PCM materials (Zhang et al., 2019). In addition, PCM was encapsulated with disulfiram (DSF) and photothermal Cu_2_-xSe nanoparticles (Figures 2G–I), thereby creating a photo-triggered cascade release nanoreactor (Pan et al., 2024). The fate of PCMs is explained sequentially. Typically, these PCM-based nanodelivery platforms administered in the body initially accumulate in the tumor site due to the EPR effect. Upon NIR irradiation at the predetermined tumor site, high temperature induces the phase transition of the PCM from solid to liquid, triggering the release of the encapsulated drugs. On the one hand, PCM-encapsulated nanodelivery systems prevent premature drug release and leakage of encapsulated cargo. On the other hand, PCM encapsulation enables precise drug delivery at the tumor site, depending on the light exposure time. Although these PCMs are highly effective in the triggered release of encapsulated drugs, the comprehensive explorations in terms of precise release of the encapsulated cargo and degradation kinetics of the PCMs, as well as the reproducibility on a large scale, remain to be addressed.

## 3 Inorganic nanoencapsulation

Inorganic-based components are another important class of materials that can be fabricated using various precursors, including but not limited to carbon nanotubes (CNTs), silica (amorphous, bioactive, and mesoporous), transition metal dichalcogenides, calcium hydroxyapatite, layered double hydroxides, MXenes, as well as gold-, silver-, selenium-, platinum-, and tellurium-based dots, among others (Liu C.-G. et al., 2019; Park et al., 2009). Due to extensive precursors and exceptional electronic architectures, it is feasible to fabricate several kinds of dimensional forms of materials (0-3D) and their composites (symmetric and asymmetric) based on the requirement and need for application (Liu C.-G. et al., 2019). Accordingly, these architectures offer exceptional physicochemical properties, tailorable morphological attributes, and other characteristics of autofluorescence, stimuli-responsiveness, and plasmonic resonance in biomedical applications (Xia H.-Y. et al., 2022). These inorganic-based nanodelivery systems have demonstrated remarkable efficacy in inducing tumor regression in superficial or ion-sensitive cancers. Compared to organic-based materials, the inherent non-biodegradable nature often results in the accumulation of nanoparticles in critical organs, such as the liver, spleen, and kidneys-over long-term treatments, potentially leading to chronic toxicity, oxidative stress, and inflammatory responses. Nevertheless, it has been increasingly recognized that modifying the structure would make them appropriate due to their exceptional physicochemical characteristics. In terms of applicability, these inorganic-based materials can be decomposed into ions and molecules under specific conditions, allowing the disassembling of the physical structure and molecular-level disintegration (Han et al., 2022).

### 3.1 Non-metallic inorganics

Carbon, beyond being a fundamental component of organic macromolecules essential for life, also exists in diverse forms, such as amorphous carbon nanoparticles, sp^2^ carbon nanomaterials, and diamonds. In drug delivery, carbon nanomaterials like graphene, CNTs, and fullerenes have been utilized due to their large specific surface area and ease of functionalization (Holmannova et al., 2022). In some instances, researchers have functionalized graphene oxide (GO) nanosheets with PEG, effectively loading doxorubicin (DOX) through supramolecular π-π stacking interactions (Nie et al., 2014; Biedermann and Schneider, 2016). The honeycomb lattice structure of sp^2^-hybridized carbon materials facilitated the conversion of light into heat towards exhibiting the PTT effect.

**FIGURE 3:**
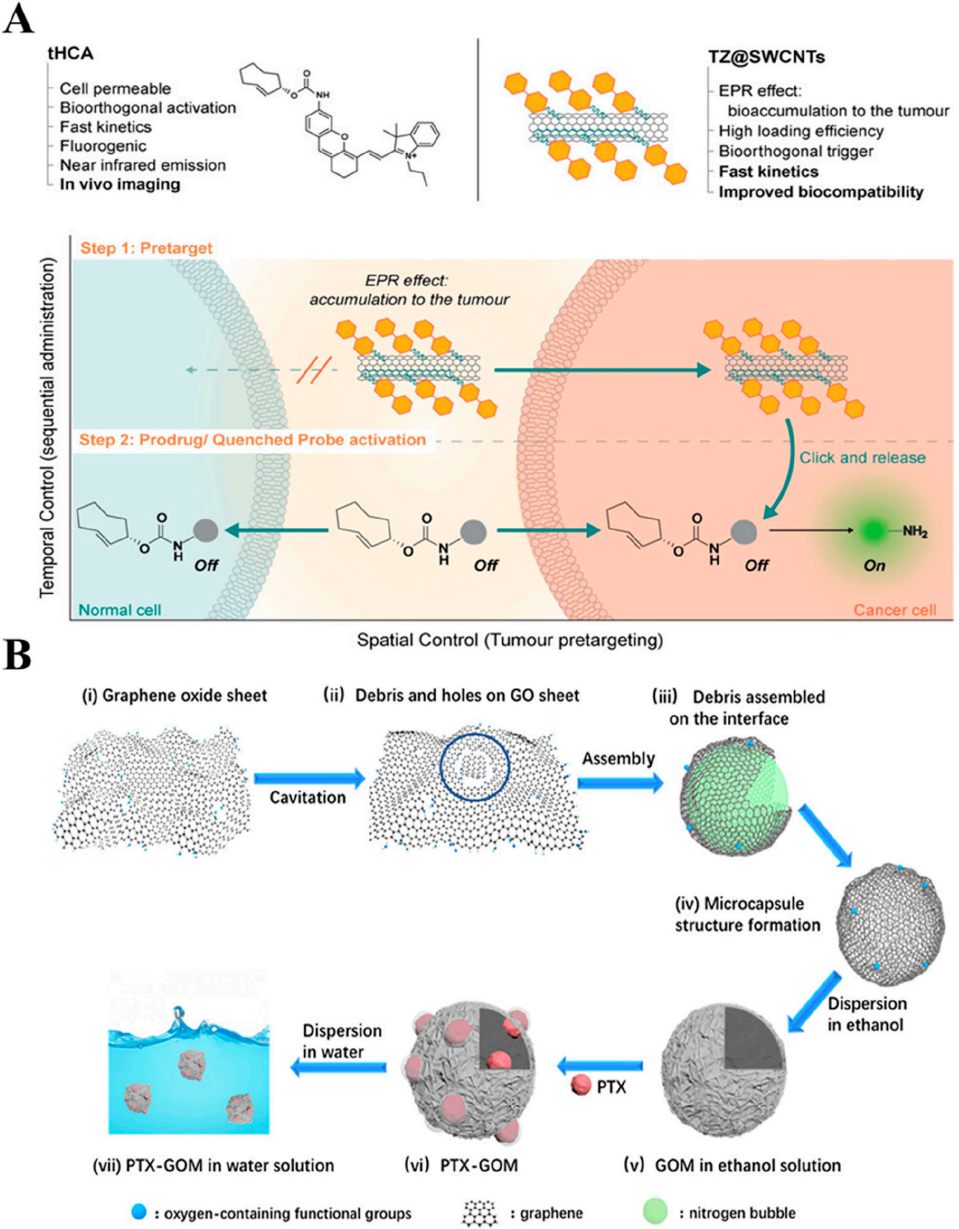
Typical non-metallic inorganic nano-drug delivery system. **(A)** Illustration of the targeted strategy for selective activation in cancer cells. The approach takes advantage of the EPR effect-enabled accumulation of TZ@SWCNTs and the bioorthogonal IEDDA cleavage reaction between tetrazine and TCO to selectively deliver active therapeutic drugs or imaging probes to the tumor. Reproduced with permission from Ref. (Li H. et al., 2020) Copyright 2020, John Wiley & Sons. **(B)** Schematic diagram of self-assembly of GOMs as a drug carrier. Reproduced with permission from Ref. (Wei et al., 2021) Copyright 2021, American Chemical Society.

Carbon materials facilitated the conversion of light into heat towards exhibiting the PTT effect. CNTs offer various advantages, such as high specific surface area, ease of internalization, and biocompatibility. Moreover, these CNTs can provide scope for functionalization in both interior and exterior, loading various drug molecules for cancer therapy. Specifically, cellular toxicity can be reduced by modifying biocompatible polymers on the surface of these carbon nanomaterials, such as graphene and CNTs. Secondly, *in situ* oxidation or doping with other biologically safe metals can appropriately increase the biodegradability of carbon nanomaterials and avoid long-term accumulation in the body while maintaining their good drug delivery performance to a considerable extent. In addition, effective targeted modification strategies are utilized to reduce the dose of loaded nanomedicine, while systematic *in vivo* and *in vitro* experiments are conducted to evaluate and optimize the loading dose of nanomedicine. Moreover, investigating its biocompatibility in the long term makes it more promising for clinical application. Accordingly, several efforts have been dedicated to generating single-walled CNTs (SWCNTs) and modified with multiple active moieties towards improved cancer therapy. In an instance, He and colleagues modified the surface of SWCNTs with tetrazine to enable drug delivery concerning “click-to-release” and real-time fluorescence imaging of tumors (Figure 3A) (Li H. et al., 2020). In addition, the designed GO structure can enhance the drug-loading effect. In an instance, liquid nitrogen cavitation was employed to self-assemble GO sheets into hollow microspheres, whose wrinkled surface and abundant polar groups could effectively load the hydrophobic PTX (Figure 3B) (Wei et al., 2021).

**FIGURE 4:**
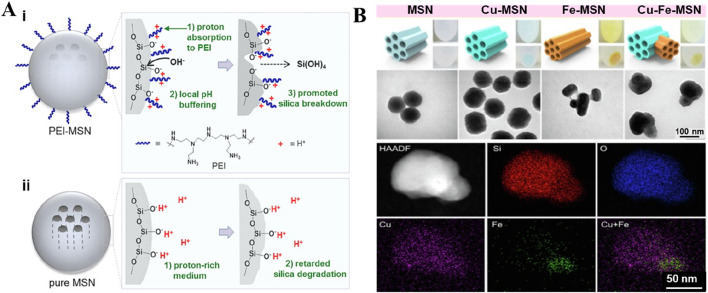
Typical non-metallic inorganic nano-drug delivery system. **(A)** Schematic depiction of the hydrolytic degradation process of PEI-MSNs (i) and pure MSNs (ii) in an acidic medium. Reproduced with permission from Ref. (Choi and Kim, 2019) Copyright 2019, Academic Press Inc. **(B)** Schematic representation as well as the morphology of the Janus-type architectures. The fabrication of various metals-doped MSNs via a simple route, along with the optical images showing the color change and TEM images revealing the morphology of different surfactant-extracted MSNs samples (bare MSNs, Cu-MSNs, Fe-MSNs, and Cu-Fe-MSNs). Elemental mapping based on TEM presents the arrangement of varying chemical species along with the incorporation of metals in the Janus-type mesoporous frameworks. Reproduced with permission from Ref. (Liu C.-G. et al., 2019) Copyright 2019, Elsevier.

Silicon dioxide (SiO_2_) nanoparticles offer enormous advantages of tailorable morphological features, colloidal stability, and biosafety. The abundant surface chemistry and internal porous structure largely ensure the diversity of drug loading capability, facilitating effective drug delivery (Huang P. et al., 2021). Nevertheless, the poor degradability of SiO_2_, resulting in harsh conditions, largely leads to the retention of bye products in the body (Kankala et al., 2020c). To promote the decomposition of particles, amorphous silica nanoparticles have been used for particle construction (Park et al., 2009). Previous reports indicated that the modification of the silica surface with polymers could promote its decomposition. In a case, silica particles were modified with polyethyleneimine (PEI), promoting degradation in both acidic and neutral environments (Figure 4A) (Choi and Kim, 2019). In several instances, our group generated the metal-doped mesoporous siliceous constructs by introducing various transition metals, specifically, divalent species (Cu, Zn, and Fe), replacing the Si atoms at the molecular level without changing the microstructure (Liu C.-G. et al., 2019; Kankala et al., 2020b). In addition, these atoms were doped in the silica framework through the coordination interactions, which not only facilitated improved encapsulation of guest species but also enabled the disassembly of the encapsulated guest species precisely in the mildly acidic environment (Kankala et al., 2020a; Kankala et al., 2020c; Kankala et al., 2022). Moreover, these coordination interactions facilitated the degradation ability of the silica framework under acidic conditions (Huang X. et al., 2021; Shi et al., 2016). Similarly, Wang and colleagues prepared the organo-inorganic hybrid disulfide bonds (S-S) doped mesoporous organosilica (MONs). These nanocomposites exhibited faster biodegradation in the simulated body fluid (SBF) mixed with GSH, attributing to the interaction between GSH and the S−S bonds in MONs. The degradation of the organic-inorganic framework could be beneficial to the effective delivery of CuPpIX to mitochondria, improving the efficiency of the sonodynamic therapy (SDT) (Wang et al., 2024c). Together, these findings suggested that it could be feasible to change the composition of the silicon source to enhance its degradation performance.

While ensuring the drug encapsulation and degradation efficiencies, the metal-doped silica particles showed a common spherical structure with single metal doping (Zhang et al., 2013). To improve the performance efficacy of the designed metal-doped silica containers, our group observed interesting findings by doping two transition (Cu and Fe) metals in the siliceous framework. Accordingly, the structure of the metal-doped silica particles was altered due to electrostatic repulsions in the siliceous framework, forming unique sphero-ellipsoid-shaped structures, such as Janus architectures (Liu C.-G. et al., 2019). As anticipated, the modified Janus-type nanoparticles could exhibit improved encapsulation of guest species, enabling their precise release in the mildly acidic environment of tumors (Figure 4B) (Liu C.-G. et al., 2019). Despite the structure, the comprehensive evaluations of safety attributes and optimization of synthesis parameters remain unexplored. In some instances, studies indicated that other irregularly structured nanoparticles showed a positive effect on cellular internalization due to the altered surface charge towards a positive end (Souri et al., 2024). The doped metals, for instance, Cu species, participate in a Fenton-like reaction, enhancing the chemodynamic therapeutic (CDT) effect of nanomaterials. Accordingly, Ca, Mg, and Zn species doped in silica presented osteosarcoma repair, promoting osteocyte regeneration and growth of bone structure in bone defect sites (Han et al., 2022).

### 3.2 Metallic inorganics

**FIGURE 5:**
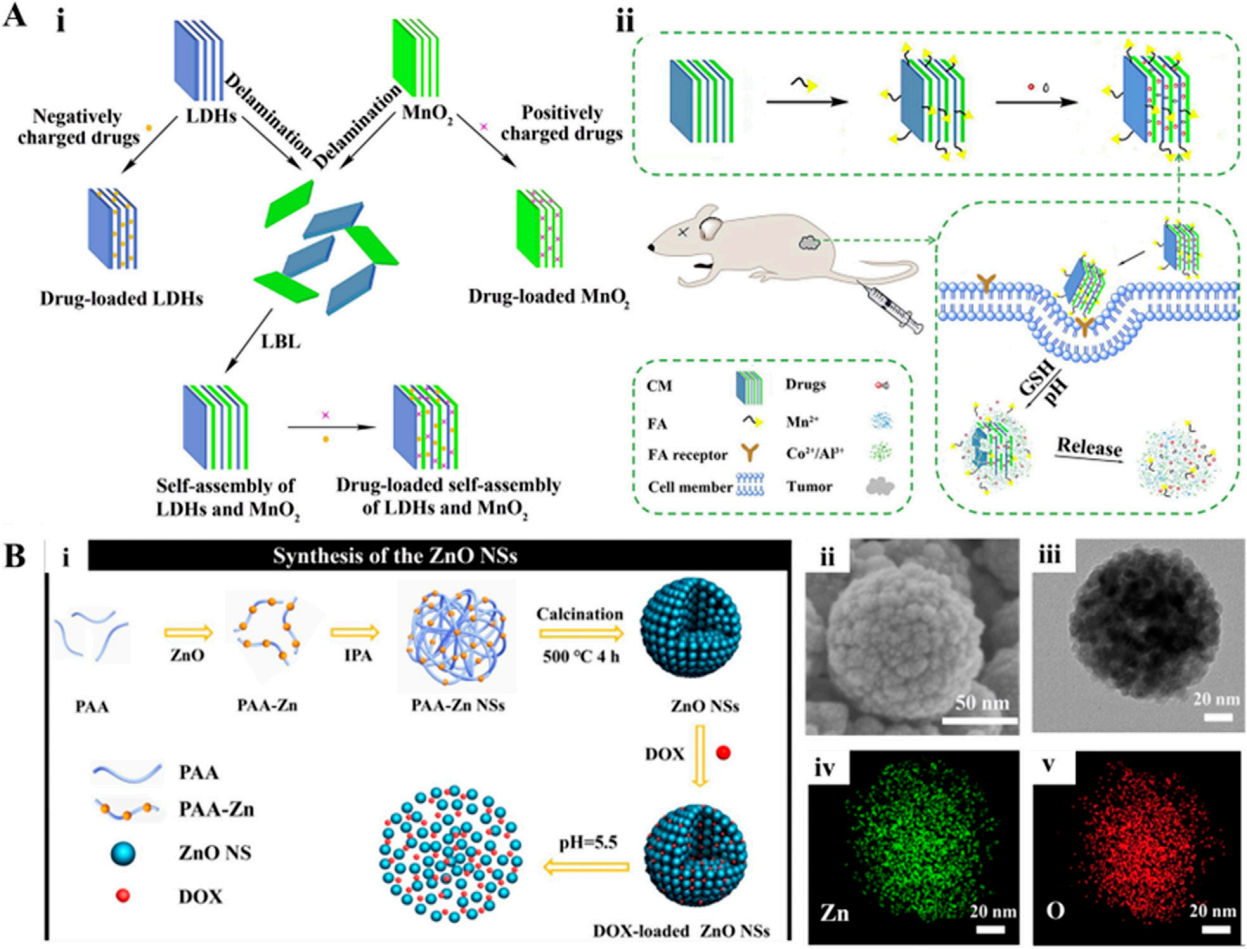
**(A)** Schematic demonstration of (i) delaminated LDHs, MnO_2_, self-assembly of LDHs, and MnO_2_ nanosheets for drug delivery; (ii) synthetic and working protocol for FA-CM. Reproduced with permission from Ref. (Wen et al., 2019) Copyright 2019, Elsevier. **(B)** Schematic illustration for the synthesis of the ZnO NSs assembled from ultrasmall particles as pH-responsive drug vehicles for cancer chemotherapy in vitro and in vivo. Reproduced with permission from Ref. (Chen et al., 2019) Copyright 2019, Elsevier.

Several metallic inorganics have been evident based on the type of precursors, offering exceptional physicochemical attributes and interesting morphological features, including layered double hydroxides (LDH), zinc oxide (ZnO)-based, manganese-based, iron-based, and copper-based constructs, with controllable shapes and surface modification capabilities. Among such metallic inorganics, LDH is one of the typical two-dimensional structured nanomaterials that possess many active sites between layers, making it extremely useful in the field of drug delivery (Kankala, 2022). Moreover, these interesting materials exhibit excellent biocompatibility and degradability, high specific surface area, pH-responsiveness, and ease of surface modification (Kankala, 2022). The surface hydroxyl groups attached to the layers not only facilitate the partial degradation of LDH in a slightly acidic environment but also enable the encapsulation of guest species, relying on the internal ion exchange of LDH to promote the release of internal drugs (Hakeem et al., 2018). Further advancements have evidenced the functionalization of LDHs by combining them with specific proteins to develop composite LDH materials for drug delivery. At the same time, the modified proteins are used to help the composite LDH materials easily penetrate the blood-brain barrier or achieve mucosal drug delivery (Wang L. et al., 2023; Wang et al., 2020). Accordingly, LDHs composed of metal cations exhibit positive charges between layers, carrying negatively charged drug molecules. To encapsulate positively charged drugs, some reports demonstrated the modification of drugs into composite drug molecules with a negative surface charge to achieve effective drug delivery (Tyner et al., 2004). In addition, several efforts have been dedicated to depositing other ions by doping and modifying the layers of LDHs using the co-precipitation method. Doping creates pores on the surface to enhance the specific surface area, enabling the encapsulation of metal nanoparticles and loading of drugs (DOX) based on hydrogen bonding interactions (Wang L. et al., 2013). To enhance the ability of LDH to carry different kinds of drugs simultaneously, Wen and colleagues self-assembled LDH with MnO_2_ nanosheets to achieve simultaneous loading of positively charged, negatively charged, hydrophobic, and hydrophilic drugs (Figure 5A) (Wen et al., 2019). Despite the success in exploring the potential of LDHs as a carrier and a shell for encapsulating various drugs and nanoparticles, further investigations are required to explore the optimization parameters as well as stability in the physiological fluids.

Among the semiconductor materials, ZnO-based materials are characterized by excellent chemical stability, biocompatibility, biodegradability, and sensing capabilities in optoelectronic applications. Typically, Zn, an essential element in the human body, plays a crucial role in cellular growth, energy metabolism, gene expression, and gene stability (Wang et al., 2024b). Due to its biocompatibility and high stability, ZnO can be utilized to deliver drugs and track materials in real time. In some instances, researchers developed the ultrasmall-sized ZnO nanoparticles self-assembled to form a structure of large nanospheres, in which the middle pores could serve as spaces for carrying drugs, for instance, DOX. These ZnO nanoparticles could effectively achieve rapid decomposition and release of drugs after cellular internalization of ZnO species (Figure 5B) (Chen et al., 2019). In addition to drug delivery, these nanomaterials could exhibit anti-tumor effects, such as the internalized ZnO nanoparticles in cancer cells reacting with lysosomes intracellularly. Further, the acidic environment in lysosomes triggered the release of Zn^2+^ ions and the generation of H_2_O_2_. Accordingly, the excessive Zn^2+^ could disrupt redox balance within cells, leading to apoptosis and cytoskeletal remodeling. The influence of high levels of Zn^2+^ was referred to as Zn^2+^ disturbance, interfering with and suppressing the mitochondrial electron transport chain (Wang et al., 2024b). These consequences could induce substantial ROS generation in mitochondria and cause oxidative stress, promoting the apoptosis of cancer cells. Moreover, researchers have functionalized ZnO nanoparticles with various surface active agents such as polyglycerol and PEG. To achieve the degradation of ZnO and pH-responsive drug release under acidic conditions, these functionalized ZnO particles could encapsulate drugs (DOX, daunorubicin, DNR) through chemical interactions (Cai et al., 2016; Yang X. et al., 2019; Wang et al., 2017; Zhang et al., 2011). Along this line, several metal-based nanocarriers have been developed, such as manganese-based, iron-based, and copper-based compounds, with controllable micro shapes and surface modification capabilities. These materials not only enable responsive drug delivery but also can decompose to release functional ions, which can further enhance their anti-tumor effects.

Among the variable valence metallic materials, iron-, manganese-, and copper-based materials offer exceptional prospects in drug delivery and tumor treatment due to their controllable microstructure, surface modifiability, as well as their inherent Fenton-based and Fenton-like reactions and specific responsive abilities. For instance, MnO_2_ offers unique advantages in cancer therapy due to the multi-dimensional structural arrangement from zero-dimensional (0D) to three-dimensional (3D) on the nanoscale of MnO_2_ (Xia H.-Y. et al., 2022). Several reports indicated that 1D MnO_2_ nanotubes could serve as effective PA imaging detection agents. Typically, MnO_2_ could degrade *in vivo* in a GSH-rich environment in melanoma, leading to the vanishing of PAI signals and distinguishing normal tissues from tumor lesions (Liu et al., 2018). Similarly, Fan and colleagues loaded the photosensitizer chlorin e6 (Ce6) onto two-dimensional (2D) structured MnO_2_ nanosheets, initially protecting Ce6 from light-induced inactivation. After being transported to cancer cells, Ce6 was degraded and released to achieve enhanced PDT effects (Fan et al., 2016). Due to their large specific surface area and ample space, the 3D structured MnO_2_ nanoparticles could be used for modifying and loading drugs as well as other 0D structured nanodots in their confined nanospaces. Notably, organic materials are wrapped around the surface of these inorganic constructs to ensure the stability of composite nanoparticles and effective drug transport, which will be introduced in the section on composite nanoparticles.

## 4 Bio-based nanoencapsulation

Although various synthetic (organic and inorganic) materials have been applied for the encapsulation of nanoparticles or drugs, most of these materials failed to assure delivery due to certain limitations, including reproducibility, lack of stability, as well as failure to cross the biological barriers and rapid clearance by the body immune system considering them as foreign bodies. To overcome these limitations, several kinds of biological components have been extracted to encapsulate the nanoparticles and their loaded drugs for improved cancer therapy, such as bacterial membranes, cell membranes, and cellular carriers (Table 1). Biomimetic nanocarriers derived from natural membranes offer unique advantages in tumor-targeted drug delivery. Yet, these biomimetic nanocarriers face significant challenges, such as complex and variable production processes, limited stability during storage, potential immunogenicity from residual endotoxins, and difficulties in achieving high drug loading and controlled release. These challenges may result in inconsistent therapeutic outcomes, limiting their clinical translation. However, recent advances in structural modifications and careful source selection have mitigated these issues by enhancing membrane stability and enabling tailored release profiles. Moreover, the integration of stimuli-responsive elements now allows these systems to disassemble under tumor-specific conditions, thereby transforming their inherent limitations into distinct therapeutic advantages.

### 4.1 Bacterial membranes

**FIGURE 6:**
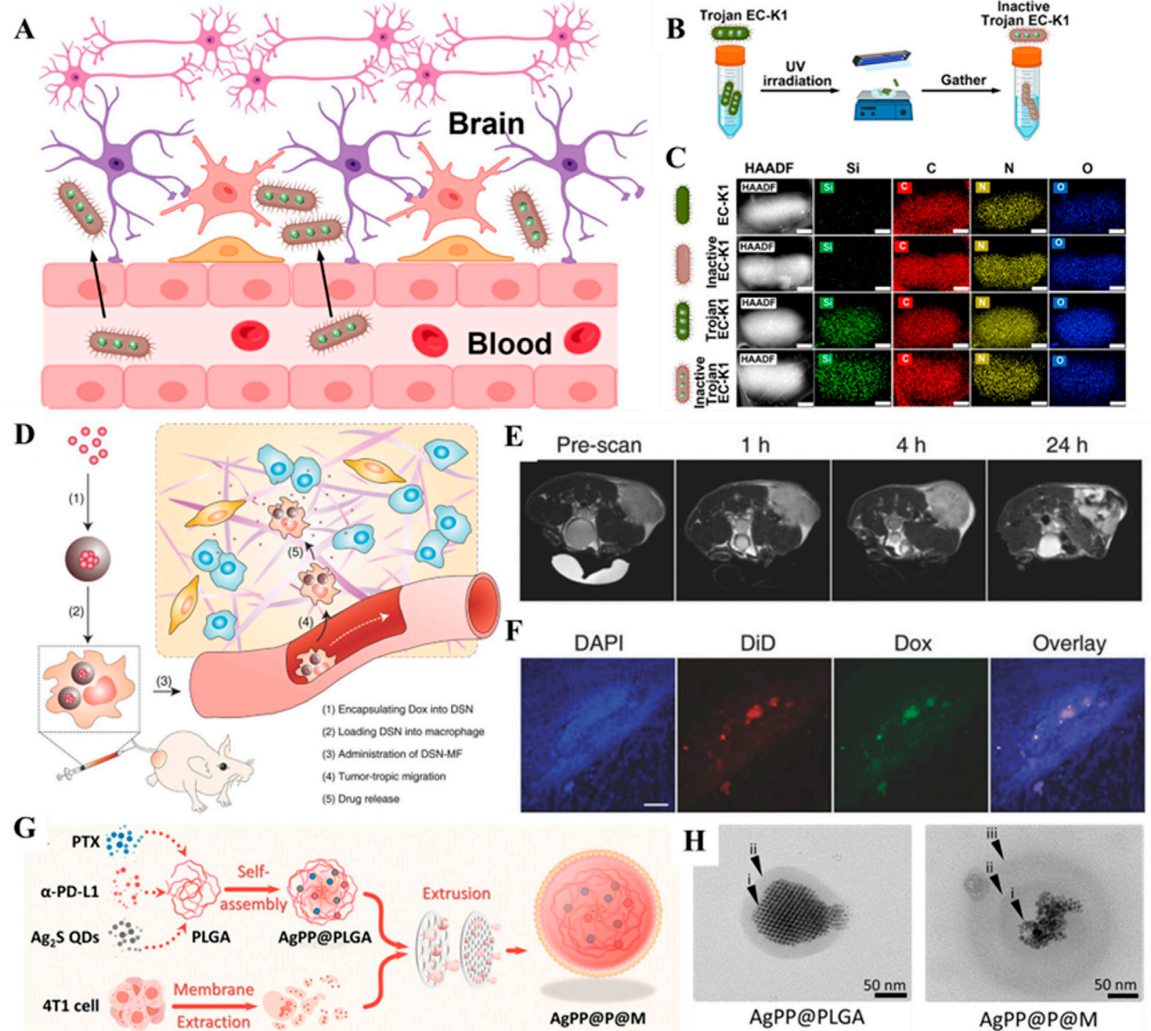
**(A)** Schematic illustration of inactive Trojan EC-K1 crossing the blood-brain barrier. **(B)** Scheme illustrating the construction of the inactive Trojan EC-K1 system. **(C)** Elemental mapping in HAADF-STEM images of EC-K1, inactive EC-K1, Trojan EC-K1, and inactive Trojan EC-K1. Scale bars: 200 nm. Reproduced with permission from Ref. (Lu et al., 2023) Copyright 2023, American Chemical Society. **(D)** Schematic illustration of nanocapsule-laden macrophages for drug delivery to tumors. **(E)** Axial T2 MR images, acquired at 0, 1, 4, and 24 h post i.v. Injection of DSN-MF cells. The cells were preloaded with iron oxide nanoparticles. **(F)** Confocal microscopy images of tumor cryosections using the z-stack scan mode (step = 2 μm). DSN-MF cells were prelabeled with DiD. Red, DiD; green, DOX; blue, cell nuclei. Scale bars, 50 μm. Reproduced with permission from Ref. (Zhang et al., 2018) Copyright 2018, John Wiley & Sons. **(G)** Self-assembly of NIR-II Ag_2_S quantum dots (QDs), PTX, and α-PD-L1 into PLGA nanoparticles, followed by encapsulation with a 4T1 tumor cell-derived membrane to construct AgPP@P@M nanoparticles. **(H)** TEM images of AgPP@PLGA nanoparticles (left) and AgPP@P@M nanoparticles (right). Reproduced with permission from Ref. (Xiong et al., 2024) Copyright 2024, John Wiley & Sons.

In recent times, the use of bacteria as carriers has emerged as an interesting area of research for delivering nanodrugs in cancer therapy. Bacteria possess unique advantages that make them ideal drug delivery tools for cancer treatment (Wu and Liu, 2022). Firstly, certain anaerobic or microaerophilic bacteria (*Salmonella*) (Chen W. et al., 2022) are naturally home to tumor tissues, especially in the hypoxic TME. Considerably, the property makes bacteria effective for targeted drug delivery, reducing damage to healthy tissues (Zhou et al., 2018). To further enhance specificity, researchers employed biomimetic targeting strategies, such as retaining bacterial adhesion proteins or chemically conjugating ligands (e.g., antibodies or folate) to the membrane surface, enabling precise recognition of tumor receptors (Gao J. et al., 2021). Through genetic engineering, researchers have dedicated several efforts to modify bacteria to carry various nanodrug platforms or directly encode and secrete anti-cancer agents. Bacteria can be engineered with a “suicide” mechanism to ensure self-degradation after drug delivery, thereby minimizing side effects. Along this line, some bacteria can activate the host’s immune system, enhancing antitumor immunity through induced inflammatory responses. To avoid excessive inflammation, advanced designs incorporated with immune-modulating elements into bacterial membranes locally suppress immunosuppressive signals while amplifying therapeutic effects (Bai et al., 2024). Immunological synergy offers potential benefits for combating tumor recurrence. For instance, Chowdhury and colleagues designed a non-pathogenic strain of *E. coli*, which could release encapsulated anti-phagocytic receptor nanobody antagonists of CD47 upon specific degradation in the TME (Chowdhury et al., 2019). The engineered bacterium could serve as a safe and localized delivery system for immune therapy agents, leading to systemic antitumor immune effects. In another instance, Lu and colleagues observed that “dead *E. coli* K1 (EC-K1)” could retain the full structure and targeting ability of live EC-K1 while losing its pathogenicity. The designed nanoplatform could safely deliver therapeutic agents across the blood-brain barrier (BBB), demonstrating its potential as a means for treating bacterial meningitis and glioblastoma in mice (Figures 6A–C) (Lu et al., 2023). With these advancements in new materials technology and increasing diversity in bacterial strains, bacterial-based drug delivery is expected to become a significant direction for future cancer therapies. Future innovations may focus on dynamic-responsive carriers (e.g., pH/enzyme-triggered drug release) or synthetic biology approaches to engineer bacteria secreting localized immune-modulators, balancing efficacy with safety.

### 4.2 Cellular carriers

Typically, insufficient bioavailability of drugs in tumors remains a major issue with cancer nanoparticle-based therapeutics due to rapid clearance. Nevertheless, cellular-based carriers, with natural immune evasion mechanisms, help them avoid elimination by the immune system, including macrophages, neutrophils, and mesenchymal stem cells (Cao et al., 2018; Zhang T. et al., 2022). In addition, these cellular carriers often exhibit tumor tropism, allowing them to migrate to hypoxic or inflamed regions within the TME spontaneously. For instance, Zhang and colleagues demonstrated the significant potential of macrophages in the delivery of anticancer drugs. These membranes could efficiently sense chemotactic signals and target tumors. Further, the experimental evidence showed that loading the representative anticancer drug (DOX) into macrophages effectively inhibited tumor growth without causing physiological toxicity (Figures 6D–F) (Zhang et al., 2018). In addition to macrophages, He and coworkers reported that loading inflammatory monocytes with self-assembled legumain-activated mertansine-conjugated poly(styrene-co-maleic anhydride) nanoparticles (SMNs) allowed active targeting of lung metastases and initiated metastasis-specific intelligent drug release for anti-metastasis therapy (He et al., 2017). The intelligent release of anticancer drugs as free molecules and drug-loaded microbubbles significantly inhibited the proliferation and migration of metastatic 4T1 breast cancer cells. Although the strategy of using cell vectors to deliver nanoparticles for tumor therapy offers many advantages, the process of mass-producing cell vectors is relatively complex, and achieving standardized production for clinical use remains a challenge. However, the future of cellular delivery of nanomedicine holds great promise.

### 4.3 Cellular membranes

Among various nanoscale delivery systems, coating nanoparticles with the extracted cell membrane (CMNPS) has attracted considerable attention due to their unique features and capabilities in cancer therapy. CMNPs are often produced by coating nanoparticles with cell membranes of specific cell types, taking advantage of the membrane’s receptor, adhesion molecules, and immune escape properties. These CMNPs allow the nanoparticles to effectively evade the immune system, prolonging the circulation time and targeting cancer cells (He Y. et al., 2024). These CMNPs can be derived from RBCs, immune cells, or cancer cells, offering unique targeting advantages. The disruptive advantages of cell membrane coating technology lie in its nature, editability, and functional expandability, among others. By utilizing the natural cell structure as a shell, this technology enables the safe delivery of nanodrugs with high biocompatibility, protecting the drugs from degradation by enzymes in the bloodstream. The application of autologous or self-derived cells, such as membranes from RBCs, macrophages, or cancer cells, has garnered enormous interest from researchers as they naturally possess immune tolerance. These intrinsic markers on the cell membranes contribute to immune escape and reduce immunogenic responses, promoting long-term circulation and improving targeted delivery efficiency (Zhao et al., 2021; Zhu et al., 2023). In addition, modifying the “do not eat me” signal marker on nanocarriers, such as the CD47 (a transmembrane protein) on the surface of exosomes, can interact with the receptor signal regulatory protein alpha (SIRP alpha), effectively inhibiting macrophage-mediated phagocytosis and further reducing immune clearance (Cheng et al., 2021).

In addition, attaching targeting molecules (antibodies or peptides) to CMNPs can further enhance their specificity for cancer cells or tumor tissues. For instance, Miao and colleagues developed dynamic RBC membrane-coated elastic PEG diacrylate hydrogel nanoparticles (RBC-ENPs), demonstrating high immunocompatibility and excellent diffusion in the tumor ECM, improving accumulation at the tumor site towards deep-seated tumors (Miao et al., 2022). In another case, Xiong and coworkers created a nanodelivery system using triple-negative breast cancer (TNBC) cell membranes. The nanoplatform utilizing the biomimetic cancer cell membranes assembled with PTX, Ag_2_S quantum dots, and programmed death ligand (α-PD-L1) substantially extended the tumor remission period and significantly reduced lung metastasis (Figures 6G,H) (Xiong et al., 2024). The disruptive advantages of cell membrane coating technology lie in its nature, editability, and functional expandability, among others. By utilizing the natural cell structure as a shell, this technology enables the safe delivery of nanodrugs with high biocompatibility, protecting the drugs from degradation by enzymes in the bloodstream.

## 5 Composite nanoencapsulation

Although various materials based on organic and inorganic substrates have been employed for drug delivery, most of these nanomaterials solely suffer from various significant limitations in nanoscale smart drug delivery, such as polarity, size, ion exchange, and chemical reactions, leading to their aggregation of inorganic nanocarriers in solution or the body, or premature drug release. To address these issues, several kinds of composite nanoencapsulation strategies have been proposed (Table 1), specifically, surface modification of inorganic nanocarriers with polymers and responsive inorganic nanomaterials. Encapsulating these inorganic carriers with biomolecules can enhance their biocompatibility and improve delivery efficacy towards augmented tumor ablation. These compatible layers help reduce the toxicity and potential inflammatory reactions of drug-loaded nanocarriers in the body. These composite nanocarriers-based systems integrate diverse materials to synergistically combine functionalities for cancer drug delivery. Nevertheless, these composites face several challenges, including complex fabrication processes, heterogeneity, and difficulties in achieving reproducible batch-to-batch consistency. Moreover, the multifaceted composition may lead to unpredictable toxicity profiles, as well as suboptimal control over drug release kinetics and biodistribution, potentially compromising therapeutic efficiency. However, recent advances in material engineering, such as refined surface modifications, incorporation of stimuli-responsive elements, and improved synthesis protocols, have become research hotspots, addressing these limitations by enhancing stability, targeting specificity, and controlled release behavior. These innovations are steadily transforming composite nanocarriers into highly promising platforms for effective and safer cancer therapy. In this section, we present discussions on these composite nanoencapsulation approaches using polymer-coated inorganics and inorganic-coated inorganic materials. Moreover, a brief note on organic-inorganic composites, i.e., metal-organic frameworks (MOFs), is presented.

**FIGURE 7:**
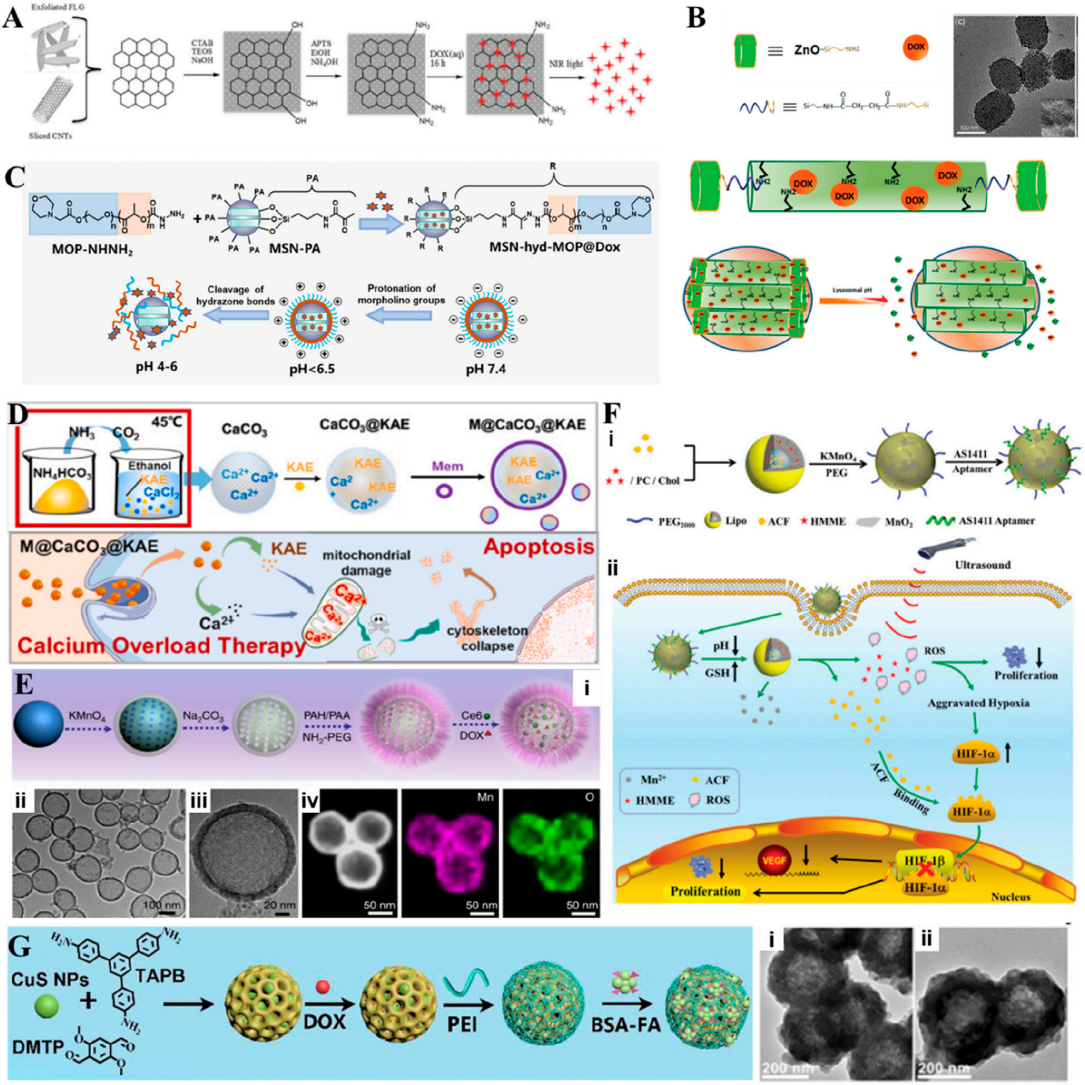
**(A)** Scheme showing the principle of the synthesis of CNT@MS or FLG@MS grafted with APTS, the loading with DOX, and the drug release actuated by T, pH, or NIR light. Reproduced with permission from Ref. (Wells et al., 2018) Copyright 2018, John Wiley & Sons. **(B)** Schematic illustration of the synthesis of ZnO@MSNs–DOX and working protocol for pH-triggered release of the anticancer drug (DOX) from ZnO@MSNs–DOX to the cytosol via selective dissolution of ZnO QDs in the acidic intracellular compartments of cancer cells, TEM micrographs of ZnO@MSNs. The inset in **(B)** is a high-resolution TEM image. Reproduced with permission from Ref. (Muhammad et al., 2011) Copyright 2011, American Chemical Society. **(C)** Schematic illustration of MSN-hyd-MOP loaded with DOX for charge reversal and controlling release. Reproduced with permission from Ref. (Yuan et al., 2019) Copyright 2019, Academic Press Inc. **(D)** Schematic illustration of M@CaCO_3_@KAE nanoparticles synthesis. Schematic illustration of M@CaCO_3_@KAE NP-mediated apoptosis. Reproduced with permission from Ref. (Li et al., 2021) Copyright 2021, Elsevier. **(E)** Synthesis and characterization of H-MnO_2_-PEG. (i) A scheme indicating the step-by-step synthesis of H-MnO_2_-PEG nanoparticles and the subsequent dual-drug loading. (ii) TEM image and (iii) magnified TEM image of H-MnO_2_-PEG. (iv) HAADF-STEM image and elemental mapping for H-MnO_2_-PEG. Reproduced with permission from Ref. (Yang et al., 2017) Copyright 2017, Springer Nature. **(F)** Scheme of Lipo/HMME/ACF@MnO_2_-AS1411. (i) Synthesis process and (ii) Schematic illustration of the proposed mechanism of Lipo/HMME/ACF@MnO_2_-AS1411 for amplification of SDT. Reproduced with permission from Ref. (Wang et al., 2018) Copyright 2018, John Wiley & Sons. **(G)** Schematic illustration of the preparation of CuS@COFs-BSA-FA/DOX. The TEM images of CuS@COFs-BSA-FA incubated in (i) water and (ii) PBS buffer (pH-7.4). Reproduced with permission from Ref. (Wang S. et al., 2023) Copyright 2023, Elsevier.

Despite exceptional bioefficacy and considerable biocompatibility, inorganic non-metallic nanomaterials often suffer from aggregation, requiring the improvement of water degradation properties. Several efforts have been dedicated to combining bacterial cellulose (BC) with carbon-based materials that can enhance the water solubility of carbon nanomaterials. In a case, the combination of BC and GO could effectively load and release DOX in a controlled manner (Zhu et al., 2015). To enhance the drug delivery efficiency of carbon-based materials, some researchers have exfoliated and coated them with mesoporous silica, creating a stable and hydrophilic layer that allows for ultra-high loading of DOX and its release under acidic conditions and laser irradiation (Figure 7A) (Wells et al., 2018). Further, responsive metallic compounds were directly grown *in situ* at the pore openings to block the pores and improve the applications of drug-loaded MSNs. Utilizing ZnO nanoparticles with pH-responsive decomposition to serve as blocking materials for larger mesoporous nanocarriers, controlling the pH-triggered release of drugs within the SiO_2_ mesopores was achieved (Figure 7B) (Muhammad et al., 2011). In addition, polymer morpholin-4-yl-acetyl-PEG-b-poly(lactic acid) (MOP) was grafted onto MSNs to prevent unintended drug leakage while facilitating cellular uptake under acidic conditions for responsive drug delivery (Figure 7C) (Yuan et al., 2019).

Calcium carbonate (CaCO_3_), as a stable and biocompatible inorganic non-metallic material like SiO_2_, not only possesses a similar porous structure but also exhibits pH sensitivity and gradual biodegradability. To achieve slow and sustained drug release in acidic environments, CaCO_3_ particles can effectively release Ca^2+^ ions. Under the influence of the TME, CaCO_3_ particles intracellularly can achieve the release of DOX and Ca^2+^ after decomposition. At the same time, CaCO_3_ particles exhibit coagulation effects in acidic blood environments in hemolysis studies, which can effectively help achieve starvation therapy at tumor sites (Zhou et al., 2023). In addition, CaCO_3_ can be easily modified on the surface with groups targeting cancer cells, achieving targeted delivery of anticancer drugs (Ren et al., 2022). The released Ca^2+^ ions have been shown to be of great significance in the treatment of calcium overload cancer, as they can lead to the destruction of mitochondrial structure and function, cytoskeletal breakdown, oxidative stress, and promote apoptosis of tumor cells (Zheng et al., 2021). To achieve this level of calcium overload treatment, a large amount of Ca^2+^ ions is required, but normal cell membranes have protein pathways that regulate intracellular and extracellular Ca ion concentrations. Therefore, it is of great significance to ensure that Ca^2+^ is not prematurely eliminated from cells after achieving effective internalization of CaCO_3_ nanoparticles. In a case, Kaempferol-3-O-rutinoside (KAE) loaded onto CaCO_3_ nanoparticles was delivered into tumor cells, disrupting the calcium balance of the cells, increasing the content of Ca ions in the cells, and achieving KAE-mediated calcium overload therapy (Figure 7D) (Li et al., 2021). In another case, CaCO_3_nanoparticles were coated with PEI, showed an intracellular proton sponge effect caused by PEI and CO_2_, not only facilitating nanomotors to escape from the lysosomes toward cytoplasm but also promoting the deep penetration and long-time retention of nanomotors inside tumor cells (Ren et al., 2022).

In addition, the inorganic metal nanocarriers used for drug delivery may induce inflammation in physiological environments, potentially leading to inappropriate interception. Biocompatible materials can be used to modify these metal compound nanocarriers to ensure stability in the body’s circulation. In a case, ZnO quantum dots complexed with PEG could form pH-responsive nanoclusters for loading large amounts of DOX (Cai et al., 2017). In other instances, constructing hollow nanocarriers, such as hollow MnO_2_ nanoparticles, designed by template method, after modification with PEG, could simultaneously encapsulated with DOX and Ce6, providing oxygen to enhance therapeutic effects during treatment (Figure 7E) (Yang et al., 2017). Various core-shell composite nanocarrier systems not only involve inorganic nanoparticles as cores with biologically compatible materials as coatings but also include structures where drugs are encapsulated with organic materials as core and then coated with inorganic materials. For instance, hematoporphyrin monomethyl ether (HMME) and acriflavine (ACF) within liposomes were encapsulated and then oxidatively synthesized MnO_2_ nanosheets on the liposome surface, followed by modification with tumor-targeting AS1411 aptamer to achieve targeted drug delivery and synergistic enhancement of SDT (Figure 7F) (Wang et al., 2018).

Developing high-performance smart nanodrug delivery systems is a promising approach. While encapsulating nanodrugs with either organic or inorganic materials alone has partially addressed the issue of circulation within the body, there is still room for improvement in tumor-specific targeting and antitumor efficacy by integrating the advantages of various materials. For example, Wang and colleagues reported a system where ultrafine CuS nanoparticles were encapsulated within engineered covalent organic frameworks. The surface of the nanodelivery platform was functionalized sequentially with water-soluble PEI and targeting molecules, such as BSA-folic acid (BSA-FA) (Figure 7G). The outer BSA-FA coating served as a specific targeting agent for FA receptors on tumor cells, enhancing the uptake of the nanoparticles by tumor cells. In addition, the hydrophilic PEI coating significantly improved the physiological stability of the nanodelivery platform (Wang S. et al., 2023).

MOFs are materials formed through the self-assembly of metal ions or ion clusters with organic ligands via coordination reactions. By altering the types of metal ions and organic ligands, various categories of MOF materials can be obtained. Due to their large surface area and ease of modification, MOF materials offer higher drug-loading capacity and more versatile targeting capabilities compared to traditional carriers. Several reports indicated that nanodrugs loaded with MOFs could significantly extend circulation time in the bloodstream (Li L. et al., 2020). Additionally, the versatility of MOF organic ligands allowed for the modification of functional molecules on their surface to achieve precise tumor targeting. In recent years, researchers have successfully utilized MOFs as carriers for targeted nanodrug delivery in experimental settings. For instance, porphyrin-based MOFs, using photosensitizers as organic ligands, could enhance PDT efficacy (Xia M. et al., 2022). Furthermore, combining porphyrin-based MOFs with upconversion nanoparticles enabled the combined treatment of hypoxic tumors. Addressing the limitations of current nanoparticle-based cancer therapeutics, Shao and colleagues designed a system that encapsulated the hypoxia-activated prodrug tirapazamine (TPZ) within the porous structure of upconversion nanoparticles (UCNP)/MOFs nanocomposites, facilitating a synergistic treatment of PDT, hypoxia-activated chemotherapy, and immunotherapy triggered by NIR light (Shao et al., 2020). In another study, Miao and coworkers encapsulated ovalbumin (OVA) within Al-MOFs and coated the surface with yeast capsules (YCs). The resulting OVA@Al-MOFs/YCs nanoparticles were used as an immunovaccine for mice, promoting the maturation of macrophages and inducing an immune response (Miao et al., 2019). Moreover, another research team utilized MOFs to load gold nanoparticles and hairpins with an outer PEG shell to enhance the biocompatibility and dispersion of the nanoplatform. This system not only facilitated the conversion of O_2_ to ROS to promote PDT but also enabled imaging of cancer-related micro-ribonucleic acid (miRNA) and dynamic monitoring of miR-21 (Yang et al., 2023). Overall, MOFs, as carriers for encapsulating nanodrugs, can overcome the limitations of single cancer therapies through functional modifications. With advancements in technology and ongoing research, MOF materials still hold significant potential in the field of nanodrug delivery for tumor treatment.

## 6 Degradation and safety

The degradation kinetics and byproducts of the designed encapsulated nanosystems critically influence their therapeutic outcomes and eventual biosafety. Typically, organic-based nanosystems, such as polymer-based carriers and liposomes, often rely on hydrolytic or enzymatic degradation. For instance, PEGylated constructs gradually undergo hydrolysis *in vivo*, facilitating the release of the encapsulated guest species, i.e., nanoparticles and their drug cargo (Meng et al., 2024). Moreover, HA-based nanoparticles are enzymatically degraded by hyaluronidase in the TME, enabling tumor-specific drug release (Gou et al., 2024). Nevertheless, the incomplete degradation of these organic constructs may trigger inflammatory responses. Similar to certain kinds of polymers, liposomal composites often rely on enzymatic (phospholipase) degradation. It should be noted that the degradation rate of these liposomal composites is influenced by membrane composition (Zhang et al., 2025). However, the degradation rate is critical, as a rapid breakdown may lead to a burst release of the drug, while a slower process may extend the therapeutic effect. If the degradation products are harmful, these composites with a slower degradation process may pose accumulation-induced potential toxicity risks. The biocompatibility and potential toxicity of these degradation byproducts are, therefore, important considerations in optimizing treatment efficacy and safety. In contrast, inorganic-based nanosystems like SiO_2_ and ZnO often exhibit pH-dependent degradation mechanisms. For instance, ZnO nanoparticles can dissolve in acidic environments to form Zn^2+^ ions (Zeng et al., 2025). Although such stability is beneficial for maintaining prolonged circulation, the incomplete degradation and long-term residue of inorganic materials may trigger inflammatory responses. These inorganic-based nanosystems may lead to accumulation-related toxicity, particularly in cases where materials remain unmetabolized (Yang G. et al., 2019). The biological membranes-based nanosystems, such as those utilizing cell membranes or bacterial vectors, show more complex degradation behaviors compared to conventional organic liposomal systems (Zeng et al., 2022; Jiang et al., 2023). Often, these slow, degradable systems are subjected to enzymatic breakdown and immune clearance. For instance, the nanosystems based on the biomimetic properties of the erythrocyte membrane can effectively accumulate in tumor tissues with immune escape and prolonged blood circulation (Qiao et al., 2022). In another instance, bacterial carriers, such as engineered bacterial outer membrane vesicles or mixed membrane vesicles containing bacterial cell membranes, can activate antigen-presenting cells while delivering multiple antigens, thereby inducing immune activation (Zhao et al., 2022). However, a large amount of pathogen-associated molecular patterns (PAMPs) is also a double-edged sword, as residual in the body may cause non-specific inflammation. Composite nanosystems integrated with multiple materials present even more intricate degradation profiles than their individual components and usually undergo staged degradation. Typically, a polymer-coated inorganic nanoparticles may first experience degradation of the outer polymer layer, subsequently releasing the inorganic core (Wu et al., 2024; Meng et al., 2024). The stability of the interface between these components plays a crucial role in determining the overall degradation behavior. The interplay of synergistic or competitive mechanisms can significantly impact drug release kinetics and the safety profile of the nanocarriers.

Another major challenge in using various nanoencapsulation strategies for effective cancer treatment is the appropriate excretion of the nanoparticles by overcoming complex physiological environmental barriers. Subsequently, the accessible clearance from the body would enable the challenge of accumulation-induced toxicity of most of the nanoparticles. Contrarily, the rapid clearance of nanoparticles through the kidneys during their conveyance through the systemic circulation is the primary challenge, affecting the targeting and delivery concentration of nanodrugs. Accordingly, rapid kidney clearance can be effectively avoided, thereby prolonging the circulation time of the nanosystems in the body (Mu et al., 2020; Xin et al., 2022). Another key physiological barrier is the recognition by the immune responses as a defense mechanism in the body. The human body comprises a complex immune system that is essentially able to recognize and eliminate allogeneic cells. Despite the advancements, the clinical development of various nanomedicines has been limited due to their high immunogenicity and poor biological stability. To this end, the biological membrane-based nanoencapsulation technology can offer superior quality over synthetic organic and inorganic-based constructs by suppressing the effector T cells and evading the recognition by the immune system (Chae et al., 2024). In addition, nanocarriers need to target the TME fully and release drugs as needed to achieve optimal biodistribution in the body. Immobilization of engineered biomolecules, such as antibodies, peptides, and aptamers on the surface of nanoparticles, can validate active targeting effects, reducing the risk of premature release or leakage of nanomedicine in other tissue microenvironments. Thus, these strategies substantially improve the accumulation and penetration of tumor areas, enhancing therapeutic efficacy and reducing biological toxicity (Gao X. et al., 2021). Although the notified targeting ligands, such as antibodies and peptides, significantly enhance the tumor specificity of nanoparticles, their stability and efficiency in dynamic *in vivo* environments still need further optimization. Future research needs to quantify targeting efficiency through multimodal imaging techniques and evaluate the impact of immunogenicity on long-term treatment. The nanoencapsulation strategy discussed in this article overcomes physiological barriers by optimizing particle size and surface properties to avoid rapid clearance, reduces immune recognition through surface modification, and employs targeted strategies to enhance biological distribution and tumor penetration. These comprehensive methods are crucial for maximizing the therapeutic potential of nanosystems in cancer treatment. In summary, considering the complexity of systemic circulation and tumor penetration trajectories, the intelligent encapsulation of nanocarriers needs to overcome the main physiological barriers of the body and exhibit well-controlled degradation behavior. Moreover, it also needs to achieve targeted drug release ability and minimal biological toxicity. Accordingly, it is critical to balance multiple aspects for appropriate delivery and safe therapy.

## 7 Future prospects and challenges

Each nanoencapsulation system presents unique advantages, limitations, and therapeutic impacts across various cancer types. Typically, the therapeutic efficacy of various nanoencapsulation (organic/inorganic/biomembrane/composite) systems varies significantly across diverse cancer types, depending on material properties and interactions with the components in the TME. Although successful in providing exceptional biocompatibility, as well as enormous encapsulation and delivery abilities, the organic-based nanosystems demonstrate moderate efficacy in deep tumors through stimuli-responsive targeting. However, the therapeutic effectiveness of the designed organic-based carriers is reduced due to the limited penetration depth in hypoxic solid tumors. The inorganic-based systems show exceptional therapeutic efficacy in superficial or ion-sensitive cancers, achieving tumor regression in preclinical models. However, they may show potential systemic toxicity in long-term treatment due to accumulation-induced toxicity risks. The bio-based systems show superior immune evasion and metastasis suppression. The therapeutic effectiveness of these nanosystems diminishes in immune-cold tumors. The composite-based nanosystems outperform others in aggressive cancers, leveraging synergistic mechanisms to address TME heterogeneity. Although the organic/inorganic systems are suitable for localized tumors, the composite-based nanosystems offer broad-spectrum efficacy, which, however, requires optimization for clinical scalability.

In general, the diversity of encapsulation materials provides additional physicochemical properties to the drug delivery nanoplatforms. For example, targeting proteins can be modified within the designed encapsulation materials to enhance the targeting capability of drug-loaded nanoparticles, facilitating better accumulation and internalization at the target site while reducing erroneous uptake at non-target locations. Magnetic encapsulation materials can aid in directing the drug delivery platform to magnetic pole regions. Thermo-responsive materials integrated into nano-carrier platforms can release drugs upon exposure to external photothermal stimuli. Biodegradable materials ensure biological safety while autonomously migrating to target locations. Additionally, materials with bioimaging capabilities can enable real-time monitoring of drug transport when incorporated into the drug delivery platform. The integration of multimodal imaging technology (magnetic resonance imaging, MRI, positron emission tomography, PET, and fluorescence, among others) provides a multi-dimensional perspective for real-time monitoring and precise control over the tracking of nanocarriers. However, the stability of radioactive labeling and the biocompatibility of magnetic materials still need to be optimized. In the future, multimodal data fusion models based on deep learning are expected to break through the technical bottleneck of single imaging and achieve closed-loop precision medicine of “imaging treatment feedback.”

Overall, the smart combination of various materials into composite materials for the modification of nanoparticle-based drug delivery platforms can not only provide internal circulation stability but also facilitate specific responsive drug release. While there have been numerous studies on the smart encapsulation of nanoparticle-based drug delivery platforms using either single or composite materials at the laboratory stage, significant challenges remain for these platforms to achieve practical application. Firstly, intelligent nanoencapsulation platforms based on organic, inorganic, and composite materials still require further exploration to achieve uniform control over the extent of encapsulation. During large-scale production, various factors, such as concentration and volume, can affect the quality of the nanoparticle-based drug delivery platform and their subsequent encapsulation process, leading to variations in their performance efficacy. Additionally, the potential accumulation of certain active materials in the body post-decomposition needs further investigation. The encapsulation of biomaterials must also consider the activity and lifecycle of cells, bacteria, and cell membranes, as the resulting smart nanoparticle-based drug delivery platforms may face challenges, such as low yield, short shelf-life, the need for on-site preparation, and susceptibility to failure. Regarding the promotion of practical clinical application, regulatory agencies in different regions have different requirements for drug certification. For example, the US FDA and the European Medicines Agency (EMA) have not unified the evaluation standards for the safety of clinically approved drugs, and they still need a large step to promote the smart nano-encapsulated drugs to clinical application (Table 2).

**TABLE 2:** A summary of various representative smart nanocarriers encapsulated, currently approved, and under trial in clinical practice.

Species	Nanoplatform	Indications	Features	Approval	References
Liposomal nanocarriers	Doxorubicin Liposome (Doxil/Caelyx)	Ovarian cancer, multiple myeloma, HIV-associated Kaposi’s sarcoma.	PEGylated long-circulating liposomes reduce cardiotoxicity.	1995 (FDA)	Zhan and Wang (2018), Zhang et al. (2022b)
	Daunorubicin Liposome (DaunoXome)	HIV-associated Kaposi’s sarcoma.	Targets tumor tissues via liposomal delivery of daunorubicin.	1996 (FDA)	Boafo et al. (2022), Alrbyawi et al. (2022)
	Irinotecan Liposome (Onivyde)	Metastatic pancreatic cancer (combined with 5-fluorouracil and leucovorin).	Prolongs drug half-life and enhances tumor drug concentration.	2015 (FDA)	Gan et al. (2025), Juang et al. (2024)
	Vyxeos (Daunorubicin + Cytarabine Liposome)	Acute myeloid leukemia (AML)	Fixed-ratio liposomal formulation improves efficacy and reduces drug resistance.	2017(FDA)	Liu et al. (2022a), Krauss et al. (2019)
Albumin-bound nanoparticles	Albumin-Bound Paclitaxel (Abraxane)	Breast cancer, pancreatic cancer, and non-small cell lung cancer.	Utilizes albumin receptor (gp60) for tumor targeting; No premedication for hypersensitivity required.	2005 (FDA)	Pascual-Pasto et al. (2022), Hassan et al. (2023), Le Large et al. (2021)
Polymeric micelles	Genexol-PM (Paclitaxel Micelle)	Breast cancer, lung cancer.	Solvent-free formulation reduces allergic reactions.	South Korea	Gupta et al. (2021), Lin et al. (2024)
	Paclical (Paclitaxel Micelle)	Ovarian cancer	Nanomicelles may accumulate in tumor tissues through the EPR effect.	Europe (selected countries)	Bernabeu et al. (2017)
Other nanocarriers	NBTXR3 (Hensify)	Soft-tissue sarcoma	Activated by radiotherapy to release high-energy electrons	EU (2020)	Chajon et al. (2019)
	mRNA-LNP (Cancer Vaccines)	Currently used in COVID vaccines (e.g., Moderna/Pfizer) and cancer vaccines (e.g., mRNA-4157) are in clinical trials.	mRNA encoding tumor antigens is delivered into cells via lipid nanoparticles to activate specific T-cell and antibody responses, enabling targeted elimination of cancer cells.	2020 (FDA, EMA)	Bevers et al. (2022), Qu et al. (2025)
Investigational nanocarriers (Not Yet Approved)	CRLX101	For refractory solid tumors.	Camptothecin polymer nanoparticles	—	Krasner et al. (2021)
	BIND-014	Prostate cancer trials	PSMA-targeted paclitaxel nanoparticles	—	Autio et al. (2016)
	Liposomal siRNA	Patisiran	Approved for non-cancer indications; cancer applications under study	—	Adami et al. (2011)

With the rapid advancement of nano-drug delivery technology, the development of smart nanoencapsulation drug delivery techniques is also progressing swiftly. Simultaneously, the field of precision oncology is constantly developing. Accordingly, *in vitro* screening platforms based on patient-derived tumor scaffolds provide new directions for the design of personalized nanocarriers. These carriers can adapt to patient-specific ECM characteristics for targeted strategies. In addition, recent research has evidenced that Artificial intelligence (AI) models can integrate tumor tissue transcriptome data with nanomedicine-related physicochemical property parameters to predict optimal nanocarrier parameters, such as targeted ligand types and drug release rates. We anticipate that the next-generation of nanoencapsulation platforms will integrate self-assembled structures and supramolecular host-guest chemistry to achieve programmed drug loading and stimuli-responsive release. Meanwhile, biomimetic materials will enhance tumor-targeting efficiency and immune escape. Combining material innovation with AI-driven design tools is expected to develop a dynamic patient adaptive system that can self-optimize drug proportion and release kinetics in real-time and promote the innovation of precision tumor therapy.

## 8 Conclusion

In summary, this article has primarily introduced smart nanoencapsulation drug delivery technologies based on different types of materials. These intelligent nanoencapsulation techniques could enhance the initial nano-carrier platforms by allowing for a greater drug load or enabling the simultaneous encapsulation of drugs with varying physical properties. The specific configurations of encapsulation technologies facilitated the responsive release of drugs under physical or biochemical conditions while ensuring stability in the circulation environment. For instance, organic systems (polymeric nanoparticles and liposomes) offer high drug encapsulation and flexible release profiles. In contrast, diverse inorganic systems often exhibit stable structures with sustained release but face challenges in complete biodegradation and potential toxicity. Bio-based strategies, including those based on cell membranes or other biological materials, display excellent biocompatibility and targeted efficacy. Yet, their loading capacity and release kinetics can be variable due to inherent biological complexity. Future encapsulation technologies utilizing different materials are expected to provide targeted solutions to the challenges currently faced, facilitating effective delivery of nano-drugs under various application conditions and achieving the transition from laboratory to clinical application.
